# Fungal Endophyte: An Interactive Endosymbiont With the Capability of Modulating Host Physiology in Myriad Ways

**DOI:** 10.3389/fpls.2021.701800

**Published:** 2021-09-30

**Authors:** Sohini Sarkar, Abhijit Dey, Vinay Kumar, Gaber El-Saber Batiha, Mohamed A. El-Esawi, Michał Tomczyk, Puja Ray

**Affiliations:** ^1^Department of Life Sciences, Presidency University, Kolkata, India; ^2^Department of Biotechnology, Modern College of Arts, Science and Commerce, Savitribai Phule Pune University, Ganeshkhind, Pune, India; ^3^Department of Pharmacology and Therapeutics, Faculty of Veterinary Medicine, Damanhour University, Damanhour, AlBeheira, Egypt; ^4^Botany Department, Faculty of Science, Tanta University, Tanta, Egypt; ^5^Departament of Pharmacognosy, Medical University of Białystok, Białystok, Poland

**Keywords:** fungal endophytes, host plant, photosynthesis, growth, nutrient uptake, stress

## Abstract

Endophytic fungi ubiquitously dwell inside the tissue-spaces of plants, mostly asymptomatically. They grow either intercellularly or intracellularly in a particular host plant to complete the whole or part of their life cycle. They have been found to be associated with almost all the plants occurring in a natural ecosystem. Due to their important role in the survival of plants (modulate photosynthesis, increase nutrient uptake, alleviate the effect of various stresses) they have been selected to co-evolve with their hosts through the course of evolution. Many years of intense research have discovered their tremendous roles in increasing the fitness of the plants in both normal and stressed conditions. There are numerous literature regarding the involvement of various endophytic fungi in enhancing plant growth, nutrient uptake, stress tolerance, etc. But, there are scant reports documenting the specific mechanisms employed by fungal endophytes to manipulate plant physiology and exert their effects. In this review, we aim to document the probable ways undertaken by endophytic fungi to alter different physiological parameters of their host plants. Our objective is to present an in-depth elucidation about the impact of fungal endophytes on plant physiology to make this evolutionarily conserved symbiotic interaction understandable from a broader perspective.

## Introduction

The indispensability of fungal endophytes in shaping plant communities is already evident following discoveries of their profound effects on plant ecology, fitness and evolution (Clay and Holah, 1999; Brundrett, 2006). With the progressive disclosure of multiple beneficial effects imposed by endophytic fungi on their host plants, the intimate relationship between plants and endophytes has received considerable attention. Fungal endophytes are capable of influencing overall plant growth by regulating photosynthesis, nutrient absorption, phytohormone levels, and other biophysiological mechanisms (Rozpądek et al., 2015; Vergara et al., 2017; Li et al., 2018). The majority of the reports (Rozpądek et al., 2015; Wang et al., 2016; Hill et al., 2019; Bilal et al., 2020) to date have documented that the impact of endophytic fungal partners supports the survival of their host plants by improving several physiological parameters of plants which promotes growth and fitness. This assistance of endophytic fungi is being utilized to increase the yield and productivity of economically important crops (Wu et al., 2019; Bononi et al., 2020). Most importantly, the potential of these endosymbionts to exert effects on hosts has also been demonstrated under various hostile conditions when most physiological processes of plants are supposed to be compromised (Xia et al., 2016; Shahabivand et al., 2017). Fungal endophytes have exhibited excellent capacity in aiding their host plants under adverse conditions inflicted by drought, salinity, heat, heavy metal toxicity, etc. (Khan et al., 2011a,b; Hamilton and Bauerle, 2012; Bilal et al., 2020). These endophytes have also proven their efficiency in increasing the resistance of their hosts against biotic stress agents like herbivory and pathogen infestation (Rodriguez et al., 2009; Bastias et al., 2017). The immense capability of fungal endophytes in restoring the fitness of plants from the effects of extreme conditions offers a huge prospect of exploiting endophytic fungi as a measure to protect crops from the damage caused by global climate change (Ismail et al., 2018; Bilal et al., 2020). A large number of reports have documented the impact of endophytic fungi on the plants by observing changes in measurable physiological parameters (Waqas et al., 2012; Wu et al., 2019). But, the investigation of the underlying mechanisms of the visible outcomes has received least attention. How fungal endophytes drive changes at molecular and biochemical levels to modulate overall plant physiology is still very obscure. Therefore, the correlation between biochemical phenomena and visible changes in plant physiological parameters under colonization with endophytic fungi needs further explanation. The tactics employed by fungal endophytes to manipulate host physiology have not been elucidated in detail in the literature available till date. Moreover, the conception acknowledged so far are scattered in different experimental reports published by scientists working in diverse fields. Hence in this review, we target to present an elaborated discussion about the physiological basis of the interaction between plants and endophytic fungi by assembling all the information from current knowledge at one place. This review encompasses the opinions of a large number of scientists who have worked on different types of fungal endophytes residing in different plant species under diverse environmental conditions. It summarizes different experimental results and the possible physiological basis of the observations assumed as causal to the result from numerous articles so that the similarity and differences between different opinions propound a complete idea of current understanding of the matter. This article tries to establish a link between all the verified and hypothesized biochemical, biophysical, molecular mechanisms employed by fungal endophytes to manipulate different host physiological processes. The goal of our review is to provide a better understanding of the interaction between plants and fungal endophytes with emphasis on the effects conferred on plant by its fungal partner and to broaden the scope of further research on this topic in myriad fields of science including ecology, agriculture and evolutionary biology.

## Role of Fungal Endophytes on Photosynthesis in Plants

### Alteration in Photosynthetic Parameters of Plants Under Endophytic Colonization

Photosynthesis is the most important physiological phenomenon for a plant’s survival. This is a crucial step for producing the primary fuel required in all the metabolic processes of plants. So, the existence of a plant is entirely dependent on the procedure of carbon assimilation through photosynthesis. The rate of photosynthesis depends on various external factors like irradiation intensity, temperature, water availability, nutrient availability, etc. Fungal endophytes which are intimately associated with plants are capable of modulating various photosynthetic parameters. But, in which way they exactly mediate the communication between different external factors and observable photosynthetic parameters is what makes us curious. Hence, our first target is to understand how the intimate symbiotic relationship of plants with fungal endophytes poses effects on the output of photosynthesis from the literature published to date. Actually, there are limited studies comparing the photosynthetic capacity of endophyte-infected and non-infected plants and those reports do not concur with each other. We have summarized those reported results in Table 1. The table depicts the discrepancies between different experimental results and it makes us more inquisitive to know how the physio-chemical processes linked to photosynthesis are coordinated altogether under the endophyte-infected conditions leading to the measurable changes.

**Table 1:** Modulation of various host photosynthetic parameters by different fungal endophytes.

Fungal endophyte	Host plant	Altered photosynthetic parameters	References
*Neotyphodium coenophialum* (Morgan-Jones and Gams)	CB1 strain of Tall fescue (*Festuca arundinacea* Schreb)	Increase in P_n_	Richardson et al., 1993
*Neotyphodium coenophialum*	DN7 Strain of tall fescue	Decrease in P_n_	Richardson et al., 1993
*Neotyphodium coenophialum*	13 Genotypic varieties of Tall fescue	Higher P_n_ at temperatures >35°C	Marks and Clay, 1996
*Colletotrichum musae* (Berk. & M.A. Curtis)	Banana (*Musa acuminata* Colla)	Decrease in maximum photosynthetic capacity	Costa et al., 2000
*Fusarium moniliforme* (J. Sheld)	Maize (*Zea mays* L.)	Decrease in maximum photosynthetic capacity	Costa et al., 2000
*Neotyphodium*	Arizona fescue (*Festuca arizonica* Vasey)	Decrease in P_n_	Morse et al., 2002
*Neotyphodium lolii* (Latch, M. J. Chr. & Samuels)	Perennial ryegrass (*Lolium perenne* L.)	Lower P_n_ at higher light intensities	Spiering et al., 2006
*Ustilago esculenta* Henn.	*Zizania latifolia* Turcz	Higher P_n_ and greater chlorophyll content	Yan et al., 2013
*Epichloe typhina* Pers. (Tul. & C.Tul)	Orchard grass (*Dactylis glomerata* L.)	Increase in P_n_ and chlorophyll content	Rozpądek et al., 2015

### Biochemical and Molecular Influence of Endophytic Fungi on Host Plant Photosynthesis

Inspite of years of intensive research on plant-fungal endophyte interactions little is known about the underlying physiological mechanisms. We get some idea from the works and interpretations of the scientists (Table 1). Richardson et al. (1993) obtained different photosynthetic responses from two different strains of tall fescue (*Festuca arundinacea* Schreb) under colonization by *Neotyphodium coenophialum* (Morgan-Jones and Gams); they correlated this phenomenon to variation of internal CO_2_ concentration found in each genotype. Costa et al. (2000) analyzed their own result into more depth. By comparing the maximum photochemical yield (F_v_/F_m_) they had found a significant decrease in the photosynthetic capacity of both endophyte-infected banana (*Musa acuminata* Colla) and maize (*Zea mays* L.). This drop of photosynthetic capacity in *Fusarium moniliforme* (J. Sheld)-infected maize was justified with a concomitant reduction in their total chlorophyll content. But there was no decrease in the chlorophyll content of banana plants infected with *Colletotrichum musae* (Berk. & M.A. Curtis) yet they showed reduced photosynthetic capacity and carbon assimilation. For searching a proper correlation they analysed the light response curve and observed that carbon assimilation in *C. musae*-infected banana decreases in low light conditions. In low irradiance, photosynthesis is dependent on the capacity of electron transport to regenerate NADPH and ATP, as light intensity increases photosynthesis becomes bounded by the carboxylation capacity of Rubisco (Dietz and Heber, 1984, 1986). Considering this fact Costa et al. (2000) opined that the endophytic fungi poses some kind of inhibitory effect on the electron transport chain that results in reduced photosynthesis in infected banana plants. They proposed that in infected bananas the fungus might have produced a toxic compound that uncoupled electron transport without causing any negative effect on chlorophyll content. The decrease in photosynthesis in the experiment of Morse et al. (2002) suggests that reduced leaf vapour conductance in infected plants may be a cause of their lower P_n_. Moreover, their chlorophyll fluorescence parameters indicated a little impact of endophyte infection on biochemical limitation of P_n_. Spiering et al. (2006) observed no difference in apparent photon yield between *Neotyphodium lolii* (Latch, M.J. Chr. & Samuels)-infected and uninfected perennial ryegrass (*Lolium perenne* L.). Hence, they interpreted that the fungal endophytes had no impact on light interception and conversion mechanism of photosynthesis. On the other hand, they noted that light-saturated, i.e., CO_2_ limited P_n_ was largely impaired by infection of endophytes. So, they conjectured that the endophytes affected CO_2_ fixation rather than light interception and photochemistry of the photosynthetic machinery. Here the action of endophyte-expressed glucanases and invertases (Lam et al., 1995; Moy et al., 2002) might have elevated the concentration of monomeric sugars in plant tissues. Scholes et al. (1994) suggested that this probably posed some inhibitory effect on the activity of Calvin cycle enzymes. Although, the concentration of the Rubisco enzyme was not altered by this action. Their experiment did not show any significant difference in transpiration rate between infected and uninfected plants. This indicated that there was no contribution of stomatal conductance on net photosynthesis rate. Hence, alterations in leaf biochemistry seemed to be the most appropriate explanation for endophyte-induced effects on P_n_. They also found that there was almost no effect of endophytes on the P_n_ of old plants. Hence, they suggested that the plant growth phase or plant age might have a role in determining the degree of endophytic impact on host plant physiology. Enhancement of P_n_ by alteration of plant biochemistry under endophytic colonization was also reported by Yan et al. (2013). They conducted an experiment by infecting *Zizania latifolia* Turcz with the endophytic smut fungus *Ustilago esculenta* Henn. and observed higher P_n_ and greater chlorophyll content in the endophyte-colonized plants than in colonization-free plants. This increase in P_n_ was attributed to the endophytic effects on the non-stomatal processes. They interpreted that elevation of maximum carboxylation rate of Ribulose bisphosphate carboxylase/oxygenase or Rubisco and maximum rate of RuBP (Ribulose 1, 5-bisphosphate) regeneration in the endophyte-colonized plants were the key factors behind the promotion of photosynthetic efficiency in them. From the work of Rozpądek et al. (2015) a much-elaborated picture of the physiology underlying endophytic fungus-infected photosynthetic enhancement can be obtained. Their experimental results showed the effect of *Epichloe typhina* Pers. (Tul. & C.Tul) on both the stomatal and non-stomatal or biochemical processes of photosynthesis in their host orchard grass (*Dactylis glomerata* L.). Almost all the photosynthetic parameters were increased in infected plants compared to the non-infected ones. The effective quantum yield in endophyte-colonized orchard grasses was increased by 23–43% with respect to the endophyte-free plants. The rate of CO_2_ carboxylation was also improved in the plants colonized by *E. typhina*. Their decreased CO_2_ compensation point supported this observation by indicating higher internal CO_2_ concentration. The concentration of total chlorophyll content was significantly (33%) increased in endophyte-colonized *D. glomerata*. The amount of chlorophyll-b in those plants was doubled changing the chl a/chl b ratio from 4:1 to 2:1. There was also a twofold increase in the abundance of PSII protein D1 and a 3-fold increase in Lhcb3 chl a/b binding proteins. Besides, the content of PSI proteins was also elevated in *E. typhina* colonized plants. This promotion of the above-mentioned components clearly indicates an overall increase in light-harvesting capacity of the colonized host plants which may improve photosynthetic efficiency in them. The electron transport efficiency through PSII was also shown to be elevated in *E. typhina* colonized orchard grasses. It suggests better utilization of solar energy for photochemistry. There was also a threefold increase in the activity of the enzyme NADPH-MDH in the endophyte-infected orchard grasses. Hence, they assumed that the malate synthesized by this enzyme could serve as an additional energy source for the elevated energy demand of the endophyte-colonized plant. This malate could serve as an energy source of the endosymbiotic fungi living in the apoplast of the plant.

From the above discussion, we get quite a detailed overview of the influence of fungal endophytes on photosynthesis and its associated physiology in the host plant. But further research is required to discover the molecular signalling mechanisms underlying the chemical crosstalk between the two organisms.

## Impact of Fungal Endophytes on Nutrient Uptake by Plants

Fungal endophytes are a large community that includes a variety of species each with different morphology, physiology and hence varying symbiotic relationships with their respective hosts. But nearly all of the studies (Vergara et al., 2017; Hill et al., 2019; Wu et al., 2019), have unanimously agreed that most endophytic fungi aid in the growth promotion and survival of their respective hosts. This capability of the endophytes hints at their authority at the stage of nutrient uptake as well as its localization in the host plant body. Considering the importance of a process like nutrient absorption we can expect the involvement of a large number of different fungal endophytes in it.

### Root Endophytic Fungi: The Major Nutrient Absorbing Fungal Endophytes of Plants

To analyze the function of endophytic fungi in nutrient uptake we must begin with fungal endophytes associated with the root, the chief nutrient absorbing organ of a plant. The existence of many root endophytic fungi have been documented to date which exhibits active involvement in aiding nutrient absorption and nutrient allocation to different plant parts. Dark Septate Endophytic (DSE) fungi, *Serendipita* (=*Piriformospora*) *indica* (Sav. Verma, Aj. Varma, Rexer, G. Kost & P. Franken), *Trichoderma* sp. etc. are some important names among them. In this particular sect of mycological research Dark Septate Fungi (DSE) have garnered much attention in recent times because of their growth-promoting effects on different species such as conifers, grasses, cabbages and others (Jumpponen and Trappe, 1998; Usuki and Narisawa, 2007; Lukešová et al., 2015). They are often compared to arbuscular mycorrhizal fungi (AMF) for showing almost equal efficiency in nutrient-receiving function. Reports have indicated that DSE can aid in plant growth promotion directly by enhancing the nutrient uptake; or indirectly by providing protection against abiotic stresses or by producing phytohormones and analogous substances (Schulz and Boyle, 2005; Usuki and Narisawa, 2007; Wei et al., 2016). The DSE belong to the class of ascomycetes. They represent a large group of facultative, biotrophic fungi characterized by darkly pigmented melanized septate hyphae. DSE produce microsclerotia that colonize root epidermis and cortex both intra- and extra-cellularly without causing any pathological symptoms in more than 600 plant species (Jumpponen and Trappe, 1998; Surono and Narisawa, 2017; Vergara et al., 2017). To date DSE fungi have been reported to assist in plant growth by enhancing both nutrient recovery and nutrient absorption. Literature suggest that DSE are capable of elevating the rate of absorption for two vital plant macronutrients- N and P in different plant species (Newsham, 2011; Vergara et al., 2017, 2018). Different reports have contributed distinctly to provide a transparent idea about the mechanisms followed by DSE fungi in this function. Surono and Narisawa (2017) performed an experiment to assess how DSE fungi help *Asparagus officinalis* L. acquire nutrients from organic and inorganic nutrient sources. They conducted an experiment by inoculating *A. officinalis* with 25 different isolates of *Phialocephala fortinii* (C.J.K Wang & H. E Wilcox) and found that three isolates were most effective in promoting the growth of *A. officinalis* under organic nutrient conditions. Hence, they proposed that *P. fortinii* has the ability to degrade organic N and P compounds into forms available for uptake of host plants. Their assumption was consistent with the previous studies which documented increased plant growth by DSE species- *Heteroconium chaetospira* Grove, *Pseudosigmoidea ibarakiensis* (O. Diene & K. Narisawa) and *Scolecobasidium humicola* under the supply of leucine and valine as organic N sources (Usuki and Narisawa, 2007; Diene et al., 2013; Mahmoud and Narisawa, 2013). Surono and Narisawa (2017) demonstrated that *P. fortinii* promotes growth of *A. officinalis* by facilitating N and P uptake from organic nutrient sources. When L-leucine and L-valine were used as organic N sources the dry weight of DSE inoculated *A. officinalis* increased significantly. But in case of phosphorus, the growth of *P. fortinii*-colonized host plants increased both in case of organic and inorganic P sources. The dry weight of plants was significantly higher both when phytic acid (organic) and calcium phosphate (inorganic) were used as P sources, respectively. Therefore, they clearly demonstrated that *P. fortinii* strains aid in N and P absorption under organic nutrient supply but could not elucidate any possible mechanism underlying the transport of P and N from media to plant through the DSE. Later Vergara et al. (2017) had come to a similar conclusion through their study on tomato plants *Solanum lycopersicum* L. They performed experiments in non-sterile sand soil under greenhouse conditions by inoculating tomato plants with three different isolates of DSE fungi (A101, A104, and A105), two of which were members of order Pleosporale (A104 and A105). They provided the DSE-treated and untreated (control) plants with the organic nitrogen source – finely ground shoot biomass of *Canavalia ensiformis* (L.) and found that DSE inoculated plants show an elevated level of shoot nitrogen content along with increased height, leaf number and total leaf area compared to the non-inoculated control plants. Consequently, the DSE-treated tomato plants had higher total aboveground dry biomass in this case. But when the same experiment was done providing the plants with an inorganic N source- ammonium sulfate-^15^N the consequences were not the same. Therefore, they suggested that these DSE fungi are capable of degrading organic N, P, and C compounds enhancing the availability of macro and micronutrients to the plants. This assumption also corroborated with the previous studies where DSE fungi have shown their ability to degrade organic compounds including starch, cellulose, lipid, amino acids, urea, gelatin, and pectin under *in vitro* conditions (Menkis et al., 2004; Mandyam et al., 2010; Surono and Narisawa, 2017). Apart from N and P, the other vital macronutrient potassium (K) was also recovered by these DSE with great efficiency. The highest K content and recovery efficiency were observed for fungus A104 when fertilized with inorganic N source and all the inoculation treatments under *C. ensiformis*-^15^N fertilization. This suggests that DSE-tomato interaction is more responsive to K than other evaluated nutrients. From this finding, Vergara and colleagues assumed that these DSE fungi helped in K uptake of tomato plants through the transposition of potassium depletion zone and K interception in locations unattainable by the root surface itself. Since K acts on cell extension and N acts on leaf elongation, Vergara et al. (2017) related these findings directly to greater total leaf area leading to greater plant exposition to sunlight. Not only macronutrient absorption, but these DSE also promoted the uptake of micronutrients by their host tomato plants. In this study, the DSE inoculation led to a significant increase in Fe, Mn, and Zn content in inoculated plants indicating better use of these micronutrients in their source. Fe in plants is utilized for the production of ferredoxin and cytochrome which act as electron-carriers of photosynthesis, Zn and Mn also aid the enzymes involved in C metabolism; besides, and Mn is also responsible for the photolysis of water in PSII. Considering this whole scenario, Vergara et al. (2017) proposed that there might be an overall influence of DSE on the photosynthetic machinery of host plants which causes growth promotion of plants. From their findings and assumptions, they concluded that tomato plants inoculated with DSE fungi exhibit better uptake of both macro and micronutrients mainly under organic nutrient sources. But, the response of plants to inoculation varies with the isolate type of DSE. It is quite evident that the best growth promotion or nutrient uptake response to DSE occurs when organic N-source is supplied but the generalized occurrence of DSE in various environments indicates that they might also aid in nutrient uptake from inorganic sources as well (Usuki and Narisawa, 2007). To discover more about the contribution of DSE in nutrient uptake, Vergara et al. (2018) conducted another research on rice plants. They inoculated rice plants (*Oryza sativa* L.) with four different dark septate fungal isolates (A101, A103, A104, and A105) collected from colonized native rice of Brazilian amazon, *Oryza glumaepatula* Steud. and performed the whole experiment under controlled condition. The *O. sativa* roots were significantly colonized by all the fungi but A103 isolate exhibited the highest potential for enhancing rice plant growth with an increase in a number of tillers and amino-N, soluble sugars as well as N, P, K, Mg, S contents in the inoculated plants. A103-inoculated plants showed significantly increased root biomass and plant height (shoot biomass). The higher root biomass reflects the greater presence of lateral and secondary roots and root hairs which is a necessary morphological adaptation for efficient nutrient (mainly N and P) uptake under low soil fertility conditions. These findings were very similar to the report of Newsham (1999). In that study DSE fungus *Pythium graminicola* (Currently called *Harpophora radicicola* (Cain) W. Gams.)-inoculated grass *Vulpia ciliate* (L.) C. C. Gmel. showed increased tiller numbers, root and shoot length and dry biomass compared to non-inoculated seedlings in glasshouse condition. The study of Vergara et al. (2018) also demonstrated another important phenomenon. They found that A103-inoculated plants showed a 30% greater accumulation of N than the control plants when ammonium nitrate was applied as the N source. This indicates that higher N absorption does not necessarily depend on the presence of an organic N source. A101 and A103 inoculation led to a lower K_m_ value for NO_3_^−^ absorption indicating a higher affinity for this radical. This resulted in higher NO_3_^−^ consumption by A101 and A103-inoculated rice plants compared to the controls. The shoots, leaves and sheathes of the plants inoculated with A103 presented a 20% increase in amino-N level compared to the control. This implies a greater influx of N and increases in the potential of protein synthesis. The soluble sugar content of the root is not affected by any of the fungal isolates used in this experiment. But, inoculation with A103 causes an significant increase in soluble sugar content of shoots, leaves and sheathes in rice plants. It indicates more efficient photosynthesis in A103-inoculated plants which concurs with the conclusion of their previous work (Vergara et al., 2017). It states that DSE-plant association can present higher levels of chlorophyll and better PSII photochemistry in the inoculated plants. Vergara et al. continued their investigation with rice and the two Pleosporale isolates (A101 and A103). To find an elaborate picture, they tried to discover the underlying molecular and genomic alterations leading to higher nutrient uptake in DSE colonized plants (2019). In this study, they inoculated *O. sativa* plants with A101 and A103 DSE isolates and provided the samples with ^15^N-ammonium sulphate as the sole N source. The A103-inoculated plants showed significantly higher ^15^N recovery efficiency than the non-inoculated control plants. They observed the activity of plasma membrane-associated proton-pumps, precisely H^+^-ATPase, H^+^-pyrophosphatase (H^+^-PPase) and nitrate transporters (NRTs). PM H^+^-ATPase is the most abundant pump in the plant plasma membrane and ubiquitous to all cell types. It is a member of P-class ATPase protein family which synthesizes phosphorylated intermediate during the catalysis of transmembrane movement. They found that when the plants inoculated with A103 were exposed to 0.2mM NO_3_^−^ the hydrolytic activity of ATP on PM H^+^-ATPase was increased by 50% compared to the control. Inoculation with A103 also significantly increased hydrolytic activity of PPi on V-H^+^-PPase in presence of NO_3_^−^. In absence of NO_3_^−^, A103 inoculation increased the transcript abundance of H^+^-ATPase protein isoforms OsA1 and OsA5 by approximately 2-fold and four-fold respectively, relative to the uninoculated control plants. However, the transcripts of the nitrate transporter-OsNRT2.1, OsNRT2.2, and OsNAR2.1 proteins were not induced by fungal inoculation. The NO_3_^−^ anions probably enter the root cells by symport with two H^+^ ions generated by PM H^+^-ATPase (Sondergaard et al., 2004). There it can be incorporated into amino acids through a series of biochemical reactions catalyzed by enzymes like nitrite or nitrate reductase, glutamine synthase etc. and the nitrate ions and amino acids that are not reduced here are transported to the aerial parts where they are utilized to make the proteins and other cellular constituents (Yoneyama et al., 2003). The higher ^15^N recovery efficiency and higher expression of H^+^-ATPase isoforms can thus be correlated. The elevated ^15^N recovery efficiency in A103-inoculated plants might have resulted in increased production of H^+^-ATPase protein isoforms which in turn aids in more NO_3_^−^ uptake and ultimately leads to promotion of plant growth. We have already mentioned that sometimes DSE promotes plant growth by indirect means which include the production of phytohormones such as auxin. DSE exudates like auxins and others may also increase the biomass of roots and aerial parts of plants (Berthelot et al., 2016; Vergara et al., 2018). Hence, Vergara et al. (2019) also speculated that auxin produced by DSE and H^+^-ATPases can induce enlargement of the root surface to be colonized by the fungi leading to greater absorption of nutrients and water. The significance of DSE in nutrient uptake becomes more prominent when they enhance N uptake in the angiosperms of the warming Antarctic maritime ecosystem as their only root colonizing fungi. In majority of terrestrial plants, symbiotic relationship with mycorrhizal fungi plays the key role in the acquisition of limiting macronutrients like N and P (Smith and Read, 2008). But, mycorrhizas are absent from the roots of vascular plants found in maritime Antarctica (Upson et al., 2008; Newsham et al., 2009). The areas of Antarctic maritime are extensively covered by mosses like *Chorisodontium aciphyllum* (Hook.f. & Wilson) Broth., which are considered to be the primary producers of this ecosystem (Hill et al., 2019). As the low temperature is unfavourable for decomposition, moss growth results in the accumulation of large amounts of soil organic matter which mainly contain D-amino acids (Wichern et al., 2004; Royles and Griffiths, 2015). The undecomposed organic nutrient material stored here for millennia is gradually mineralizing and becoming bioavailable due to the rise in temperature. But it is mostly available deeper than 25cm inside the moss bank. The root of the vascular plants that grow in this moss bank cannot itself penetrate to that depth inside the layer of moss to absorb the optimum nutrients. But when the roots are associated with DSE fungi they can penetrate deep into the moss bank and exploit the ancient nutrients stored there (Hill et al., 2019). Not only physically but the DSE also helps in N uptake chemically by producing proteolytic enzymes (Upson et al., 2009). These enzymes can mineralize peptides and amino acids in the rhizosphere making inorganic N more readily available to roots. Two native angiosperms of this region, *Deschampsia antarctica* Desv and *Colobanthus quitensis* (Kunth) Bartl. have their roots colonized by a range of DSE fungi (Upson et al., 2008; Newsham et al., 2009) which help them to acquire various forms of N (Hill et al., 2019). The report of Hill et al. (2019) indicated that DSE colonization significantly aids these two Antarctic angiosperms to acquire N as peptides of D-amino acids and L-pentapeptides which are not even recognized as viable sources of N for plants.

Apart from DSE, there are many more root endophytes the have proved their efficiency in nutrient uptake for the host plant. Pedraza et al. (2020) reported about two other root endophytes involved in facilitating nutrient absorption of the native terrestrial plants of the Antarctic. They isolated two root endophytes, *Penicillium chrysogenum* Thom. and *Penicillium brevicompactum* Dierckx. from *C. quitensis* and *D. antarctica*, *respectively*. Both of these endophytes displayed amylase, esterase, protease, cellulase, hemicellulase, phosphatase and urease enzymatic activity at low temperature. Moreover, they increased the rate of N mineralization and total biomass in colonized *C. quitensis* and *D. antarctica* plants. Considering their exolytic enzymatic activities Pedraza et al. (2020) concluded that these *Penicillium* endophytes can cause the breakdown of different nutrient sources making them available to the Antarctic plants. Consequently, it improves the growth of these plants and adaptability to the Antarctic terrestrial ecosystem.

*Serendipita indica* is another important root endophytic fungus involved in nutrient acquisition. It belongs to the class Basidiomycota, family Sebacinaceae. Sherameti et al. (2005) demonstrated that *S. indica* promotes the growth of *Nicotiana tabacum* L. var. Samsun NN (tobacco) and *Arabidopsis thaliana* (L.) Heynh. seedlings and causes large scale transfer of nitrogen from agar plates into the aerial parts of the seedlings. It is also capable of promoting growth in a fashion similar to mycorrhizal symbioses by increasing P uptake (Shahollari et al., 2005). According to the report of Shahollari et al. (2005) it enhanced P uptake by two-three folds in *A. thaliana* seedlings. *S. indica* colonization has also exhibited a significant increase in biomass and phosphorus content of maize plants (Yadav et al., 2010). It has also been reported to enhance growth in *Cunninghamia lanceolata* (Lamb.) Hook., an important forest tree species of southern China (Wu et al., 2019). Under low P conditions, *S. indica* elevated N and P uptake by *C. lanceolata* with a concomitant increase in its biomass and P allocation in leaves. In which way *S. indica* helps in the uptake of macronutrients and improvement of the nutritional status of the host has been explained from different physiological and molecular perspectives. Yadav et al. (2010) presented the functional analysis of a gene encoding phosphate transporter (PiPT) protein in *S. indica*. The PiPT is a member of high-affinity phosphate transporter family (Pht1) which is expressed on the external hyphae of the fungus associated with the root of its host. Their observation suggested that PiPT actively participates in P transportation and the presence of PiPT transcripts is 18-fold higher in the external hyphae than in internal hyphae; hence they interpreted that external hyphae are the initial site of phosphate uptake from the soil. Their study revealed another interesting fact about this phosphate transporter; observing the presence of cAMP- and cGMP-dependent protein kinase phosphorylation sites in PiPT they postulated that unlike other fungal and plant phosphate transporters, PiPT involves regulation by cAMP and cGMP-depended downstream signalling pathway. Moreover, the expression of PiPT depends on the availability of phosphate outside the cell and there is no influence of intracellular phosphate concentration on its expression. PiPT present in *S. indica* increased the P content as well as the biomass of colonized maize plants. Its growth-promoting activity was more prominent in low phosphate condition (2-fold) relative to high phosphate conditions (1.2-fold); therefore the conclusion was that *S. indica* has the ability to increase the biomass of its host plant particularly under low phosphate conditions. To explain the massive N uptake by *S. indica*-colonized tobacco and *A. thaliana* seedlings Sherameti et al. (2005) demonstrated the relation between high N uptake and its efficient metabolism. They found that in the host plant, *S. indica* increases the expression of genes encoding nitrate reductase and starch-degrading enzyme glucan-water dikinase (SEX1) along with increasing N accumulation in roots. By demonstrating the increase in NADH dependent nitrate reductase enzyme upon colonization with *S. indica* they proved that nitrogen is recruited by endophytic fungi as nitrate. This is what makes endophytic fungi different from AMF which recruits nitrogen as ammonium from the soil. Wu et al. (2019) had observed that *S. indica* colonization increased the root length of *C. lanceolata* under low phosphate condition. Under high phosphate treatments colonization by *S. indica* significantly increased phosphorus and nitrogen levels in roots. Contrarily, N and P levels in leaves were increased by *S. indica* under low phosphate treatment. They related the increased P uptake and growth of *S. indica*-colonized plants to the influence of auxin and other indole acetic acid derivatives of the fungus. The auxin produced by *S. indica* is required for its successful biotrophic colonization within the root under low phosphate levels. After colonization, *S. indica* increases auxin level in the root which promotes root hair growth and overall root development. The developed root expresses more phosphate transporters and its increased surface area interacts with diverse rhizobacteria which altogether enhance uptake of N and P. At the same time the fungus produces a large amount of phosphatases which can solubilize soil phosphorus reserves making P available for the uptake of plants. Then with the increased expression of nitrate reductase and other metabolizing enzymes, the absorbed nutrients are efficiently assimilated and allocated to the leaves of the colonized host plant. This increases the photosynthesis rate ultimately leading to greater biomass accumulation.

Apart from basidiomycetes some root endophytes of the phylum Ascomycota also have nutrient uptake potential Fungi of the genus *Trichoderma* are one of them. They have been one of the most studied filamentous fungal endophytes involved in improving the production and development of several crop species. They can be considered as one of the most representative inhabitants of soil and plant roots. *Trichoderma* strains belong to the phylum Ascomycota, subdivision Pezizomycotina, class Sordariomycetes and order Hypocreales. These fungal strains have garnered much attention for their multifaceted beneficial activities on host plants. *Trichoderma* strains can be used to control soil-borne plant diseases as well as to enhance nutrient uptake of their host plants. Their ability in facilitating the absorption of various nutrients has been documented by many scientists. De Santiago et al. (2009) reported that *Trichoderma asperellum* (Samuels, Lieckf. & Nirenberg) can ameliorate iron deficiency in white lupin (*Lupinus albus* L.) by producing siderophores which chelate iron molecules (Fe^3+^) and act as Fe donors to the plant. The study by Altomare et al. (1999) revealed that some *Trichoderma* strains can solubilize high amounts of phosphate and many complex forms of nutrient (MnO_2_, Zn and rock phosphate). Singh et al. (2019) found that *Trichoderma ressei* (Simmons.) is capable of ameliorating nutrient deficiency in rice cultivar Heena (drought resistant) and Kiran (drought-sensitive) under 50% nutrient limiting conditions. In *T. ressei*-treated Heena Mo, Zn, Cu, Co, Fe, and Mn levels were higher compared to the controls. In Kiran, *T. ressei* caused an increase in only Fe, Co, and Cu contents. The N level was non-significantly higher in both *T. ressei*-treated cultivars relative to the non-treated controls. Further, their results showed that *T. ressei* increased IAA level in the infected rice cultivars which indicates that the fungi help the plants in nutrient uptake *via* the formation of lateral roots and root hairs. They suggested that the enhanced micronutrient uptake in plants treated with *Trichoderma* occurs due to the presence of a cysteine rich cell wall protein in *Trichoderma*. This protein plays a key role in the formation and elongation of lateral root along with root hairs resulting in an increase in the total absorptive surface. It ultimately leads to higher nutrient uptake and translocation (Samolski et al., 2012). Besides, *Trichoderma* contains microbial siderophore binding sites which not only chelate Fe^3+^ ions but also bind to divalent Cu^2+^, Pb^2+^, Zn^2+^, Cd^2+^, Ni^2+^ ions, trivalent Al^3+^, Mn^3+^, Co^3+^ ions and some tetravalent ions like Pu^4+^, Th^4+^ (Peterson et al., 2004). Bononi et al. (2020) isolated *Trichoderma* strains from the Amazon rainforest and demonstrated their capability to mineralize/solubilize phosphate resulting in the growth of soybean plants (*Glycine max* L.). Fungi can occupy larger spaces and ranges in soil than bacteria and produce many organic acids which play an important role in solubilizing inorganic phosphate (Sharma et al., 2013). The participation of *Trichoderma* in plant waste decomposition hints at their capability of mineralizing organic nutrients as well (Brotman et al., 2013). These fungi can also produce organic acids and phosphatase enzymes to solubilize soil phosphate into di- or mono-basic phosphate ions which can be readily absorbed by plants. But the amount of phosphatase produced by the fungi depends on the variety of fungal strains and source of phosphorus present. Bononi et al. (2020) had applied *Trichoderma* in combination with two different P sources – rock phosphate and triple superphosphate. Observing higher phosphatase secretion in combination with rock phosphate they postulated that rock phosphate has more insoluble phosphate in it which needs greater enzymatic activity to become mineralized, hence it induced the fungi to produce more phosphatase. On the other hand, triple superphosphate has a higher content of soluble phosphate available to the plant; so it does not require greater phosphatase activity. Their report clearly indicated that application of *Trichoderma* with a phosphorus source enhance P-uptake by soybean plants leading to promotion of their growth.

**Figure 1:**
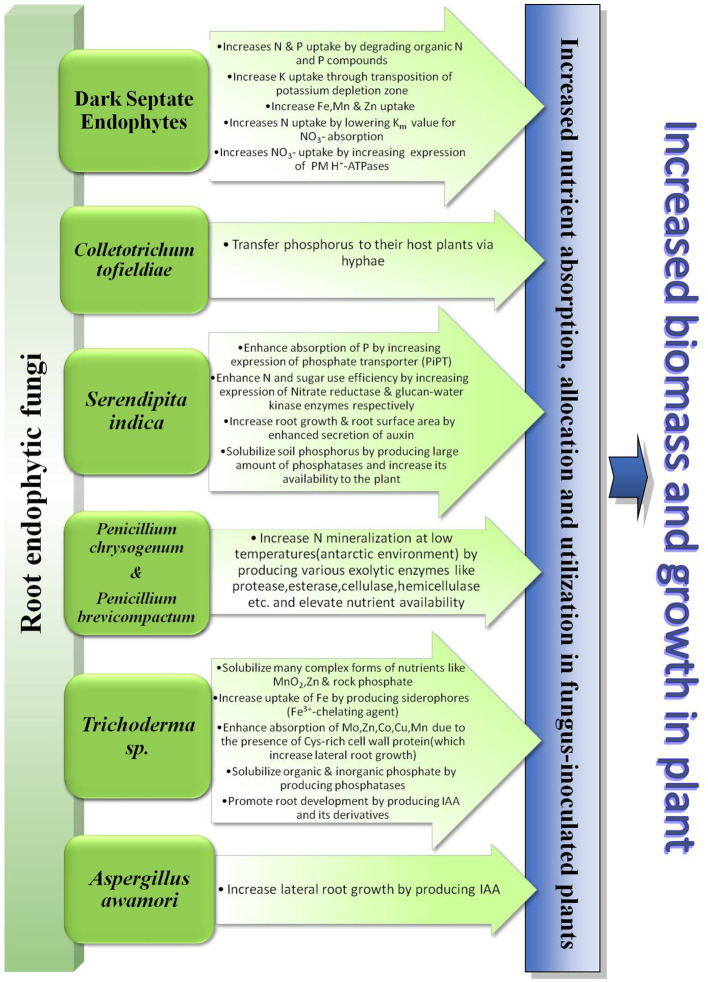
Diagrammatic presentation of the role of root endophytic fungi in plant nutrient uptake.

There have been reports on many other root endophytic fungi belonging to the class Ascomycota. *Colletotrichum tofieldiae* (Pat.) Damm, P.F. Cannon & Crous. and Heliotale Fungus F229 are two such endophytes that are endemically found in *A. thaliana* and *Arabis alpina* L. respectively. These two fungi transfer phosphorus to their respective hosts *via* their hyphae and promote plant growth under low P conditions (Hiruma et al., 2016; Almario et al., 2017). Hiruma et al. (2018) conjectured that non-mycorrhizal Brassicaceae plants benefit by symbiotic association with endophytic fungi of genera *Cladophialophora*, *Periconia*, *Colletotrichum* etc. under low P condition. Mehmood et al. (2019) demonstrated the growth-promoting activities of the endophytic fungi *Aspergillus awamori* Nakaz. w11. This endophyte enhanced growth in maize plants by producing IAA. The IAA produced by the fungi helps it to colonize maize roots and therefore promote nutrient absorption by increasing lateral root growth of the plant. In Figure 1 we have demonstrated the different ways employed by all these root-associated fungal endophytes to enhance nutrient uptake by host plants.

Hoysted et al. (2019) demonstrated that fine root endophyte (FRE) Mucoromycotina, an ancient fungal lineage supplies their vascular host plant *Lycopodiella inundata* (L.) Holub. with increased N in exchange for C from the plants. Mucoromycotina FREs are considered to form a symbiotic association with non-vascular plants; but the study of Hoysted et al. (2019) demonstrated that Mucoromycotina provides its vascular host, *L. inundata* with 145 times more N than their nonvascular hosts. This result clearly proves the ability of Mucoromycotina in assisting nutrient uptake of hosts and the ancient lineage of these two mutualists indicates how the symbiotic relationship has been evolutionarily conserved.

### Fungal Endophytes Involved in Nutrient Acquisition Need Not Be Exclusively Root-Associated

Root endophytes may take the lead role in absorbing minerals for the host plants but the contribution of some fungal endophytes has also been documented which are not exclusively root endophytes. They can inhabit both root and aboveground plant parts or may be exclusively associated with aerial parts. *Phomopsis liquidambari* (C.Q. Chang, Z.D. Jiang & P.K. Chi) is one of them. It is a common endophytic fungus found in melon, cantaloupe, eggplant, watermelon, atractylodes, mangrove plants, rubber trees etc. (Li et al., 2010; Shishido et al., 2010; Rocha et al., 2011). In Table 2 we have summarized the mechanisms employed by this fungal endophyte to promote nutrient uptake. The table shows the direct mechanisms which are employed by *P. liquidambari* to enhance nutrient uptake and subsequent growth in host plants. But, Chen et al. (2011) reported the potential of *P. liquidambari* in aiding plant growth indirectly by degrading phenolic acid allelochemicals released by decomposing foliage. 4-hydroxybenzoicacid (HBA) is a common phenolic acid allelochemical released by decomposing plant residues (Inderjit and Nilsen, 2003). Its phytotoxic effects include inhibiting seed germination and root growth by arresting the activities of metabolic enzymes in plants. *P. liquidambari* (strain B3) can utilize 4-BHA itself for its growth (Chen et al., 2011). Hence, they are capable of degrading this phytotoxic chemical leading to decreased accumulation of soil phenolic acids which indirectly results in plant growth promotion (Dai et al., 2010).

**Table 2:** Different mechanisms employed by *Phomopsis liquidambari* for increasing nutrient uptake in plants.

Host plant	Alteration in nutrient absorption	Underlying physiological mechanism	References
Rice (*Oryza sativa* L.)	Increased N acquisition and metabolism especially under low N condition	(i) Increase NO^3−^ and NH_4_^+^ absorption and NR and GS activity	Yang et al., 2014
Rice	Improved N availability and utilization under low N conditions	(i) Enhance root development increasing IAA, CTK, ETH levels in plants.	Li et al., 2018
		(ii) Increase NR and GS activity.	
		(iii) Upregulate N metabolism related genes (OsNR1, OsGS1, OsGS2, and OsNADH-GOGAT).	
		(iv) Increase expression of ammonium and nitrate transporter genes.	
Peanut (*Arachis hypogaea* L.)	Enhance Fe and Mo uptake	(i) Increase expression of Fe and Mo-related genes (AhFRO1, AhIRT1, and AhMOT1).	Su et al., 2019
		(ii) Produce low molecular weight organic acid which increase Fe and Mo availability in the rhizosphere by acting as a metal chelating agent.	

There are also some exclusively aerial tissue-inhabiting fungal endophytes that aid in plant nutrient uptake. These groups of endophytes mainly include various species of *Epichloe* which are common symbionts of the members of *Poacae* family in which they inhabit the above-ground tissues. Table 3 shows the contributions of these endophytes in improving nutrient absorption.

**Table 3:** Improved nutrient uptake by various endophytic fungi which are not root-associated.

Fungal endophyte	Host plant	Alteration observed in nutrient acquisition	References
*Epichloë festucae*	*Festuca rubra* L.	Increases P and N content in shoots	Vázquez-de-Aldana et al., 2013
*Epichloë* sp.	Perennial ryegrass (*Lolium perenne*)	Increases Mn content	Soto-Barajas et al., 2016
*Epichloe gansuensis* (C. J. Li & Nan.) Schardl.	Drunken horse grass [*Achnatherum inebrians* (Hance) Keng.]	Increases N and P content	Wang et al., 2018
*Colletotrichum tropicale* (Rojus, Rehner & Samuels)	Cacao tree (*Theobroma cacao* L.)	Increases ^15^N uptake in at whole-plant level	Christian et al., 2019

### Fungal Endophytes as Potential Biofertilizer

Analyzing various scientific reports we conjecture that fungal endophytes are highly capable of promoting plant growth and survival by enhancing nutrient absorption through various pathways. Not only nutrient absorption but also nutrient allocation and utilization can be modulated by fungal endophytes as per the need of their hosts. The mechanisms followed by various endophytic fungi to enhance nutrient uptake includes altering root morphology, upregulating the synthesis of nutrient transporter proteins as well as producing different secondary metabolites to chelate and solubilize nutrients (Yadav et al., 2010; Li et al., 2018; Su et al., 2019). Published literatures confirm the efficiency of fungal endophytes in this particular function in various plant species including crop plants like rice, peanuts, maize, soybean, etc., which hints at the huge prospect of exploiting these fungi as potential biofertilizers in agricultural fields. But, different endophytic fungus adopt different pathways to promote growth of its host plant. Hence, to ascertain the optimum growth of plants applying a combination of fungal endophytes to a single plant species can be opted. For this purpose the compatibility of each endophytic species with each plant and with other endophytes needs to be verified. In addition to promoting growth of plants, endophytic fungi can endow their hosts with resistance against various stress conditions which make it more ideal to be used as biofertilizer in different agricultural fields. Biofertilizer is a crucial component of sustainable agriculture and multifarious microbes like fungal endophytes offer an excellent option for the advancement of this field.

## Role of Fungal Endophytes in Mitigating Various Stresses on Their Host Plants

The principal reason that made fungal endophytes a major research interest is their ability to alleviate the effects of different stress conditions in their host plants. Due to their sessile nature plants are susceptible to various adverse conditions created by numerous biotic and abiotic sources. Reports have suggested that the challenges on plant physiology imposed by abiotic adversities like drought, salinity, alkalinity, UV-Rays, heavy metal accumulation, etc., becomes easier to overcome when the plants are in a symbiotic relationship with different fungal endophytic species (Bu et al., 2012; Hamilton and Bauerle, 2012; Leitão and Enguita, 2016; Shahabivand et al., 2017). The same efficiency of endophytic fungi has also been proven in increasing tolerance of plants to different biotic stress conditions like herbivory, pathogen infestation, etc. (Saikkonen et al., 2010; Bastias et al., 2017). These beneficial consequences indicate the prospect of using the science of this symbiotic interaction in agriculture to improve crop yield under adverse conditions created by environmental and anthropogenic causes.

### Fungal Endophytes: A Dependable Tool for Protecting Plants From the Ill-Effects of Biotic Stresses

Enhancing tolerance of plants to biotic stress conditions like herbivory, pathogen infestation, etc. influences plant physiology positively and increases plants’ fitness in an indirect manner. Fungal endophytes are broadly categorized into two major groups as clavicipitaceous and non-clavicipitaceous. Clavicipitaceous endophytes are predominantly found in grasses, whereas the non-clavicipitaceous fungi infect various vascular and non-vascular plant species (Rodriguez et al., 2009). Clavicipitaceous endophytes produce numerous secondary metabolites which endow their host plants with anti-herbivory mechanisms against various vertebrate and invertebrate herbivores (Saikkonen et al., 2010; Gange et al., 2019). Studies have suggested that clavicipitaceous endophytes evolved to plant biotrophic form through a series of inter-kingdom jumps from free-living insect-parasitic forms (Koroch et al., 2004; Spatafora et al., 2007; Torres et al., 2007). As these endophytes had descended from insect pathogens they did not possess enzymes or toxins to degrade plant tissues (Rodriguez et al., 2009). Hence, their colonization is not limited by plant defensive mechanisms and they can act as effective entomopathogenic agents from within the host plants conferring protection to them. In most cases fungal endophytes affect the physiology of herbivorous insects to protect their hosts. It includes a reduction in insect developmental rate (Akutse et al., 2013), limiting insect growth rate, reducing insect survival and oviposition (Lacey and Neven, 2006; Martinuz et al., 2012), etc. But, in some cases, endophytic fungi do alter plant physiology to some extent to make them more resistant to herbivory. This may include some basic physiological changes in plant anatomy like increasing cellulose content and lamina density in leaves to provide toughness which reduces herbivory especially by leaf-cutting ants (Khare et al., 2018). Previously, Harman et al. (2004) similarly opined that endophytes help plants to produce lignin and other cell wall components which reinforce cell wall and make it difficult for pathogens to infect. Sometimes the fungal endophytes may enhance plant immunity by promoting endogenous defence responses mediated by jasmonic acid (JA). Bastias et al. (2017) proposed that *Epichloe* endophytes undertake two different mechanisms to increase plant resistance to herbivory. They can produce fungal alkaloids and can also promote jasmonic acid (JA) pathway in the host plant. The JA dependent defences are effective against a broad spectrum of chewing insects. A probable mechanism explaining the promotion of JA pathway in plants under *Epichloe* colonization comes from the study of Johnson et al. (2003). According to their report, *N. coenophialum*-infected tall fescue showed increased expression of TFF41 protein which has similarity with ω-3 FAD enzyme of potato and parsley. This enzyme elevates the level of jasmonic acid precursor- trienoic fatty acid (Johnson et al., 2003). Atia et al. (2019) demonstrated potential antagonistic effects of *S. indica* against Root Knot Nematode (RKN) *Meloidogyne incognita* (Kofold & White) in cucumber plants (*Cucumis melo* cv. Hesham). Their study suggested that *S. indica* promotes salicylic acid (SA) levels in the root of host cucumber plants to resist RKN and mitigate severe damages caused by them in the root. Upon colonization, fungal endophytes create a barrier in host plant tissue that prevents other foreign phytopathogenic organisms from colonizing the same plant; this confers protection to plants from phytopathogenic diseases (Moy et al., 2000). *Epichloë festucae* (Leuchtm., Schardl & M.R. Siegel)-infected turfgrasses were proven to be significantly resistant over uninfected turf to two major leaf spot pathogens, *Sclerotina homeocarpa* and *Laetisaria fusiformis* (Bonos et al., 2005; Clarke et al., 2006). The mechanism of disease resistance seems to be a contribution of antifungal compounds produced in endophyte-colonized plants. But, whether these compounds are produced by the endophyte itself or by the plant in response to endophyte is not very clear. Xia et al. (2016) reported that the presence of *Epichloe* endophyte could ameliorate the damage caused by powdery mildew pathogen, *Blumeria graminis* (DC.) in *A. inebrians* plants. They demonstrated that the *Epichloe* endophyte prevents loss of chlorophyll and enhances host plant performance under pathogen stress. Fungal endophytes also take part in deterring mammalian herbivores from their host plants (Rodriguez et al., 2009). Li et al. (2004) reported about narcosis of grazing animals caused by *Epichloe gansuense* (C. J. Li & Nan.) Schardl.-infected Drunken horse grass [*Achnatherum inebrians* (Hance) Keng.]. They assumed that the toxic effect on grazing animals is probably due to the presence of two major ergot alkaloids, ergonovine and lysergic acid amide in endophyte-infected *A. inebrians*. Panaccione et al. (2006) performed experiments with plants with and without endophyte-derived alkaloids and found that rabbits consume less of the plants which contain ergot alkaloids. Hence, they conjectured that endophytes may not always be acutely toxic to mammals but have anti-feeding effects. From this discussion, it is evident that endophytic fungi are capable of antagonizing different plant pathogens and herbivores through different means but principally *via* producing secondary metabolites. This property of endophytic fungi can be exploited in making biopesticides which can be an eco-friendly replacement for chemical pesticides and fungicides used to protect plants from different antagonizing organisms.

### Increased Abiotic Stress Tolerance: A Result of Physiological Changes in Plants Driven by Fungal Endophytes

Global climatic changes are imposing new challenges on plant physiology by intensifying various forms of abiotic stresses. In this scenario fungal endophytes are boon to agriculture for their potential in increasing host tolerance to drought, salinity, heavy metal toxicity, waterlogging etc. The abiotic stress conditions negatively affects plant growth and productivity by decreasing photosynthesis, macro and micro-nutrient uptake, water absorption, stomatal conductance, and many other physiological parameters (Bu et al., 2012; Lamaoui et al., 2018; Bilal et al., 2020). There are some common physiological pathways and components in plants that are manipulated by endophytes in general for counteracting multiple abiotic stress elements simultaneously. The most important of them is antioxidant machinery which serves as the saviour of plants by attenuating the effects of oxidative stress. Oxidative stress arises in plants as a result of many hostile conditions including water deficiency, high temperature and salinity stress, alkalinity stress, (Bu et al., 2012; Hamilton and Bauerle, 2012; Lubna et al., 2018) most of which are associated with drought. The antioxidative system has been extensively studied for understanding the benefits of fungal endophytic association in drought stress resistance. The agents that bring antioxidants into action are called the Reactive Oxygen Species (ROS) which are the free electron-containing byproducts of aerobic metabolism (Hamilton and Bauerle, 2012). During photosynthesis, chlorophyll becomes excited after absorbing solar energy as photons and remains in this state until electron acceptors arrive to mediate its neutralization. When light absorption surpasses photosynthetic utilization (e.g., in case of photorespiration with high UV exposure) the equilibrium of the system is perturbed and as a result, the probability of singlet ROS production is increased (Gill and Tuteja, 2010). In normal conditions, there are an adequate amount of antioxidants in the system which scavenge ROS. But, in response to stresses such as drought, UV exposure, etc., the balance between the formation of ROS and antioxidants becomes unsettled which causes the accumulation of hydrogen peroxide (H_2_O_2_), super oxide (O_2_^−1^), hydroxyl radical (OH) that leads to damage of photosynthetic cells (Foyer and Noctor, 2011). One of the major deleterious effects of ROS accumulation is lipid peroxidation of membrane lipid leading to disruption of the cell membrane and leakage of solutes (Deshmukh et al., 1991). A prime indicator of lipid peroxidation is MDA (malondialdehyde) accumulation (Del Rio et al., 2005) which becomes very high under drought stress. To prevent these deleterious effects of ROS toxicity, higher production of antioxidative agents is required and the presence of fungal endophytes have been reported to elevate the levels of many of the essential antioxidants (Hamilton and Bauerle, 2012; Dastogeer, 2018; Sadeghi et al., 2019). The antioxidative machinery of plants consists of detoxifying enzymes like ascorbate peroxidase (APX), catalase (CAT), glutathione reductase (GR), superoxide dismutase (SOD), polyphenol oxidase (PPO), peroxidase (POD), ascorbic acid oxidase (AAO), and non-enzymatic antioxidant molecules, i.e., ascorbate (AsA), glutathione (GSH), etc. (Hamilton et al., 2010; Dastogeer, 2018). Colonization by the fungal endophytes has been found to enhance the effectiveness of the host antioxidative system by manipulating the production or activity of different antioxidants. The involvement of different fungal endophytes in improving the efficiency of antioxidative system in various plants under stress has been summarized in Table 4. Most studies conducted in this field have shown the involvement of endophytic fungi in manipulating the production or activity of the above-mentioned antioxidants only. But, the report of Khushdil et al. (2019) showed alleviation of salt stress in *Pennisetum glacum* (pearl millet) by endophytic fungus *Aspergillus terreus* Thom. The results of LC MS/MS analysis demonstrated the presence of quinic acid, ellagic acid, calycosin, wogonin, feruloylquinic acid, caffeic acid phenylethyl ester, D-glucoside, myricetin, propoxyphene, and aminoflunitrazepam in *A. terreus* inoculated plant extracts. All of these secondary metabolites were antioxidants with radical scavenging activities which had reduced lipid peroxidation in pearl millet under salt stress.

**Table 4:** Different fungal endophytes which modulate the efficiency of antioxidative system to endow resistance against abiotic stress in host plants.

Fungal endophyte	Host plant	The type of stress alleviated	Alteration in antioxidative system	References
*Serendipita indica*	Barley (*Hordeum vulgare* L.)	Salt (NaCl) stress	Increased CAT and APX activity	Baltruschat et al., 2008
*Serendipita indica*	Chinese cabbage (*Brassica campestris* ssp. *Chinensis*)	Drought stress	Decreased MDA level and increased SOD, POD activity	Sun et al., 2010
*Neotyphodium* sp.	Sleepy grass (*Achnatherum robustum* (Vasey) Barkworth)	Drought stress	Increased GR activity	Hamilton and Bauerle, 2012
*Neotyphodium* sp.	Arizona fescue (*Festuca arizonica* Vasey)	Drought stress	Increased APX production	Hamilton and Bauerle, 2012
EF0801	Rice (*Oryza sativa* L.)	Heavy metal (Pb stress)	Increased activity of SOD and decreased MDA content	Li et al., 2012
EF801	Rice	Alkalinity (Na_2_CO_3_) stress	Reduced MDA content and increased CAT, POD activity	Bu et al., 2012
*Trichoderma hamatum* (Bonord.) Bainier	*Ochradenus baccatus* Delile.	Salt (NaCl) stress	Decreased MDA content and increased activities of CAT, POD, APX, GR, and SOD	Hashem et al., 2014
*Trichoderma harzianum*	Indian mustard (*Brassica juncea* (L.) Czern.)	Salinity (NaCl) stress	Decreased MDA content and increased GST, POD, and GR expression	Ahmad et al., 2015
*Trichoderma harzianum* (Th-56)	Rice	Drought stress	Increased SOD, POD, and GR activity	Pandey et al., 2016
*Exophiala pisciphila* H93	Maize (*Zea mays* L.)	Heavy metal (cadmium) stress	Reduced MDA content and increased SOD, CAT, POD activities, and GSH content	Wang et al., 2016
*Trichoderma harzianum* (Th-6)	Maize and Rice	Salinity (NaCl) stress	Increased CAT and SOD activity	Yasmeen and Siddiqui, 2017
*Aspergillus flavus* Link.(CHS1)	Soybean (*Glycine max* L.)	Salinity (NaCl) stress	Increased activity of SOD and CAT	Lubna et al., 2018
*Penicillium citrinum*, *Aurobassium pullunts*, *Dothideomycetes* sp.	Mandarin plants (*Citrus reticulata* L.)	Drought stress	Elevated the levels of AsA and GSH and increased activities of CAT, SOD, APX, and GR	Sadeghi et al., 2019
*Paecilomyces formosus* (LH10), *Penicillium funiculosum* (LH06)	Soybean plants	Heavy metal, high temperature, and drought (HTD) stress	Decreased MDA content	Bilal et al., 2020
*Aspergillus foetidus* (AdR-13)	Soybean and Sunflower (*Helianthus annuus* L)	Heat stress	Increased GR, POD, CAT, SOD, and AAO content	Ismail et al., 2021

Reports quite evidently indicate the involvement of fungal endophytes in enhancing the activity of antioxidative machinery in plants under stress conditions. But, exactly how they elevate the level of different antioxidants has not been investigated or explained by most of the literature. Tanaka et al. (2006) had documented the capability of fungal endophytes in producing ROS but they did not study its impact on their hosts. White and Torres (2010) hypothesised that these fungi produce ROS for oxidizing and denaturing host cell membrane so that nutrients leak from the cell into the apoplast where the fungal hyphae can absorb them for the fungus itself. So, it is possible that the fungi produce ROS for themselves which eventually act as a secondary signalling agent to induce antioxidant production in plants. Contrarily, Wang et al. (2016) hypothesized another possibility. From their experiment they concluded that a DSE named *Exophiala pisciphila* McGinnis & Ajello., regulate genes encoding antioxidant enzymes for providing protection against ROS in maize plants under Cd stress. These are just a few possible mechanisms which can be employed by fungal endophytes to induce the antioxidative machinery. But, there is lack of confirmed evidences for these probable mechanisms. Hence, to understand the exact molecular and biochemical modulations behind this effect further research is needed.

Proline and flavonoids are among the other important stress-reducing agents of plants which have also been reported to be manipulated by fungal endophytes under stress (Khan et al., 2011b, 2013). Proline is an amino acid that acts as an osmolyte and a buffering agent for cellular redox that alleviates the negative impacts of free radical activity in plant cells under stress (Kishor et al., 2005; Kaushal et al., 2011). But, the accumulation of proline seemed to be controversial as it was not clear whether its accumulation provides greater stress tolerance or acts just as an indicator of stress injury (Ashraf and Foolad, 2007). This controversy still prevails as the experimental reports do not seem to concur with each other. Khan et al. (2013) found significantly higher proline content in *Penicillium resedanum* McLennan, Ducker & Thrower., LK6-inoculated pepper (*Capsicum annuum* L.) plants under heat stress. They conjectured that increasing proline content in stressed plants was a strategy of endophytic fungus *P. resedanum* to confer protection from the stress effects. Song et al. (2015) concluded in a similar way. They Observed significantly higher proline content in *Epichloë*-inoculated Wild barley (*Hordeum brevisubulatum* (Trin.) Link.) under waterlogging stress and subsequently resistance of these inoculated plants against waterlogging was increased. Contrastingly, Ismail et al. (2018) observed significantly lower amount of proline in *Aspergillus japonicus* Saito., EuR-26-associated soybean and sunflower (*Helianthus annus* L.) plants under high temperature (40°C) and opined that endophyte helped reducing stress severity in host plants which resulted in subsequent decline of stress symptoms like proline. Flavonoids help plants to combat many abiotic stress conditions including salinity (Khan et al., 2011b), cold and freezing (Jaakola and Hohtola, 2010) by acting as a ROS scavenging antioxidant agent (Chutipaijit et al., 2009). Khan et al. (2011b) documented significantly higher levels of different isoflavones in soybean plants inoculated with GMH-1a (A strain of *A. fumigatus*) under salinity stress. These isoflavones include aglycones, malonyl, acetyl and glucosides. Later Khan et al. (2013) also found the same increase in isoflavones (daidzin, daidzein and m-glycitin) in *P. resedanum* LK6-infected *C. annuum* plants under heat stress. They concluded that endophytic fungi elevate flavonoid levels in plants under extreme conditions to reduce the negative impacts of the stress.

Phytohormones play a crucial role in modulating plant physiology under various hostile conditions (Wani et al., 2016) and numerous reports have documented the involvement of fungal endophytes in regulating plant hormone levels in their hosts (Khan et al., 2017; Ismail et al., 2018). In this regard the most important phytohormone is abscisic acid (ABA) which is also known as the “plant stress hormone.” It exerts its protective effects by stimulating stomatal closure in order to reduce water loss and it also collectively increases the plant stress tolerance by activating many stress-responsive genes (Herrera-Medina et al., 2007; Aroca et al., 2008). ABA biosynthesis has a vital role in plants’ response to drought, UV-irradiation, heavy metal and temperature stresses (Bilal et al., 2020). Ismail et al. (2014) reported that ROS can modulate the release of ABA. The involvement of antioxidative system in endogenous hormonal regulation under stress conditions has also been reported by Hasanuzzaman et al. (2017). Under any stress condition ABA concentration in plants generally rises. But, Khan et al. (2011b) observed reduced endogenous ABA content in *Aspergillus fumigatus* Fresenius.-inoculated soybean leaves under salinity stress. They conjectured that due to the presence of the endophytic fungi the inoculated plants faced less stress compared to their control which resulted in significantly lower ABA accumulation in inoculated plants. Reduction in endogenous ABA content was also observed in cucumber plants associated with *Phoma glomerata* Wollenw. & Hochapfel., LWL2 and *Penicillium* sp. LWL3 under drought and salinity stress (Waqas et al., 2012). The same result was found by Ismail et al. (2018) in *A. japonicus*-aligned soybean and sunflower plants under thermal stress. Bilal et al. (2020) demonstrated that association with LHL06 and LHL10 endophytic fungi led to significantly lower ABA content in soybean plants under HTD stress. Furthermore, they observed that co-inoculation with LH06 and LH10 also downregulated ABA-dependent transcription factors and induced ABA-independent pathways for combating combined HTD stress. They opined that co-inoculation with the two isolates increased chlorophyll content and photosynthetic rate leading to reduced ABA content in the soybean plants. Decrease in ABA content was also found in the endophyte-inoculated Antarctic plant, *C. quitensis* under UV-B radiation (Ramos et al., 2018). UV-B is known to cause damage in plants but the endophytes probably conferred protection from UV-B by improving plant physiology which resulted in lower ABA in them. ABA is not the only phytohormone to confer protection to plants in extreme conditions. The other important plant hormones, i.e., auxin (IAA), gibberelic acid (GA), ethylene, etc., also come in rescue of plant physiology under hostile conditions (Leitão and Enguita, 2016; Wani et al., 2016). But, the crisis arrives when external stress severely reduces the biosynthesis of these hormones in the plant itself. In this situation, fungal endophytes elevate the levels of the phytohormones in plants to help them counteract the effects of stress (Khan et al., 2017; Ismail et al., 2018; Bilal et al., 2020). IAA is one of the most abundant hormones of the auxin class known for inducing cell division and elongation. Gibberellin is the classical growth-promoting hormone; however, its biosynthesis and recovery is necessary during the late phases of salt response to promote recovery (Geng et al., 2013). Different Penicillium strains have exhibited extensive capability to produce various bioactive forms of gibberelin under salinity stress conditions (Leitão and Enguita, 2016) which has been summarized in Table 5 along with the information about other phytohormone producing fungal endophytes. *A. japonicus* increased various growth parameters of soybean and sunflower plants under heat stress by producing IAA and GA (Ismail et al., 2018). Bilal et al. (2020) attributed improved growth of endophyte-associated soybean plants under combined HTD stress to the IAA and GA producing ability of LH10 and LH06 endophytes. The IAA and GA produced by these endophytes most likely increased root elongation and altered root absorption properties that helped the plants to overcome stress by gaining height and biomass. The ability to produce phytohormones makes these endophytic fungi capable of being biostimulants of plants and increasing plant resistance against heavy metal toxicity. As reported by various studies, heavy metals negatively affect plant growth and development by reducing shoot and root length, biomass production, impairing photosynthesis, water, and nutrient absorption (Guo et al., 2016; Ranieri et al., 2019). Enhancement in auxin degradation by increased activity of indole-3-acetic acid oxidase under Cd toxicity was another reason for causing decreased growth in plants under Cd stress (Chaoui and Ferjani, 2005). In this situation metal resistant endophytic fungi with GA and IAA producing capability play a vital role by increasing bio-active GA levels that inhibit DELLA proteins and consequently enhance plant growth (Hamayun et al., 2017). Waqas et al. (2014) observed improved growth in soybean plants treated with a combination of endophytic fungus-*Galactomyces geotrichum* E.E. Butler & L.J. Petersen., WLL1 and hardwood-derived biochar under Zn stress. They conjectured that the IAA-producing capability of *G. geotrichum* might be a pivotal factor in increasing the resistance of soybean plants against zinc heavy metal stress and promoting its growth. Khan et al. (2017) demonstrated that IAA-producing endophytic fungal strain *Alternaria alternata* Keissl., RSF-6L increased IAA levels and subsequent growth in *Solanum nigrum* L. plants under Cd stress.

**Table 5:** Different fungal endophytes that assist in plants’ survival under stress by producing phytohormones.

Endophytic fungi	Host plant	Phytohormone produced	Survival aid	References
*Penicillium citrinum* KACC43900	*Ixeris repens* L.	GA	Improves growth under salinity stress	Khan et al., 2008
*Penicillium funiculosum* LHL06	Soybean plants (*Glycine max* L.)	GA	Promote growth under salinity stress	Khan et al., 2011a
*Penicillium sp*. SJ-2-2	*Suaeda japonica* Makino	GA	Promote growth under salinity stress	You et al., 2012
*Galactomycesgeotrichum*WLL1	Soybean plants	IAA	Improves growth under Zn stress	Waqas et al., 2014
*Penicillium resedanum LK6*	Pepper (*Capsicum annuum* L.)	GA	Promote growth under salinity stress	Khan et al., 2015
*Fusarium sp*. CBRF44	*Brassica napus* L.	IAA	Promote growth under Cd and Pb stress	Shi et al., 2017
*Alternaria alternata* RSF-6L	*Solanum nigrum* L.	IAA	Improves growth under Cd stress	Khan et al., 2017
*Aspergillus japonicus* EuR-26	Soybean plants, Sunflower (*Helianthus annus* L.)	IAA and GA	Improves growth under heat stress (40°C)	Ismail et al., 2018
*Paecilomyces formosus* (LH10), *Penicillium funiculosum* (LH06)	Soybean plants	IAA and GA	Improves growth under combined HTD stress	Bilal et al., 2020

Not only modulation of phytohormone levels but there are also various other means by which fungal endophytes increase plant fitness under heavy metal toxicity. One common mechanism is improving photosynthesis of the host plant to induce its growth under stress (Shahabivand et al., 2017; Yihui et al., 2017). The cause underlying the enhanced photosynthesis is assumed to be the improved chlorophyll biosynthesis due to an increase in Mg and N uptake by plants associated with fungal endophytes under stress (Malinowski et al., 2000; Shahabivand et al., 2017; Bilal et al., 2020). Metal resistant fungi are capable of detoxifying the toxic metals by immobilizing them *via* the formation of insoluble metal oxalate or chelation on melanin-like polymers (Khan et al., 2017). Pietro-Souza et al. (2020) observed accumulation of the Hg mainly in shoots and roots of endophyte-infected *Aeschynomene fluminensis* Vell. and *Z. mays*, *respectively*. They opined that inoculation of plants with these fungi altered Hg translocation factor favouring its accumulation in roots and shoots; and also provided more resistance against Hg^2+^ by mediating mercury volatilization. Maize plants inoculated with *Gaeumannomyces cylindrosporus* (Hornby, Slope, Gutteridge & Sivanesan.), a DSE under Pb stress also showed altered translocation factor for Pb which caused accumulation of the metal in roots and prevented its phytotoxicity in aerial parts of the plant (Yihui et al., 2017). In this regard, Shahabivand et al. (2017) on the basis of their own results opined that chelation of Cd ions within the fungus or adsorption of Cd on the chitin of fungal cell wall led to Cd accumulation in roots and inhibited its translocation to aerial productive parts of sunflower plants. Finding significantly lower heavy metal (Cd, Ni, and Al) accumulation in roots and shoots of soybean plants inoculated with LH06 and LH10 endophytes, Bilal et al. (2020) attributed this to the down-regulation of heavy metal transporter genes. Their results indicated that co-inoculation with LH06 and LH10 downregulated the expression of GmHMA13, GmHMA14, and GmHMA18 genes which encode heavy metal transporting proteins of P-type ATPase subfamily in soybean plants. Previously, Wang et al. (2016) had also demonstrated down-regulation of a heavy metal transporter gene in endophyte inoculated plants under metal stress. Their results showed that inoculation with *E. pisciphila* significantly decreased the expression of ZIP genes in both roots and leaves of maize under Cd stress. The ZIP family genes are known to encode various metal transporters including those mediating entry of Cd^2+^. Moreover, DSE inoculation resulted in accumulation of a greater proportion of Cd as cell wall integrated/bound-Cd which prevented penetration of Cd into cells and reduced the fraction of soluble Cd in maize leaves and roots. This was another mechanism undertaken by DSE for conferring protection to maize plants against Cd toxicity. In addition to these, DSE upregulated MTP (metal tolerance protein) and PCS (phytochelatin synthase) gene expression in inoculated maize plants. Both of these genes encode proteins involved in Cd^2+^ sequestration and detoxification processes. Recently, the report of Aziz et al. (2021) showed the contribution of *Aspergillus flavus* (Ch-01) in promoting growth and alleviating toxicity in *S. lycopersicum* under Cr and Cd stress. This fungal endophyte reduced translocation of Cd and Cr from root to above-ground parts of tomato plants. In endophyte-inoculated tomato plants the relative expression of *SlGSH1* and *SlPCS1* genes, which help in metal chelation was high under the presence of Cd and Cr. Also, the production of IAA, flavonoids, phenols, proline, soluble sugars and proteins was high in tomato plants in presence of *A. flavus*.

## Conclusion

**Figure 2:**
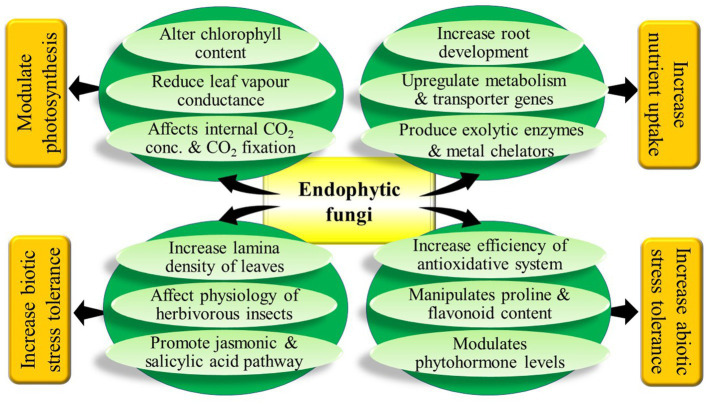
Graphical abstract.

In conclusion, it can be said that the research done to date hints at the diverse beneficial effects of fungal endophytes on their host plants (Figure 2). But, to increase the utilization efficiency of fungal endophytes in agriculture further research is necessary to explore all the costs and benefits of harbouring fungal endophytes under different circumstances. Many crop plants have been subjected to experiment with fungal endophytes but there are many more economically important plants on which fungal endophytes are yet to be tested. Endophytic fungi have been reported to be associated with almost every terrestrial plant species but very little has been explored about the interaction of all these documented endophytic fungi with plants of different taxa. Most importantly extensive research is warranted to unravel the molecular mechanisms and signalling pathways undertaken by endophytic fungi to exert their effects on plants. This is necessary for making fungal endophytes an important option to be considered in different biotechnological applications. Our review gives a holistic idea about the plant-endophytic interaction with the details available from various recent literature. It will definitely assist future research on this aspect.

## Author Contributions

SS took the lead in writing the manuscript. PR AD and VK corrected and updated it. GB, MAE and MT finalized the manuscript and arrangeded for funds. All authors contributed to the article and approved the submitted version.

## Conflict of Interest

The authors declare that the research was conducted in the absence of any commercial or financial relationships that could be construed as a potential conflict of interest.

## Publisher’s Note

All claims expressed in this article are solely those of the authors and do not necessarily represent those of their affiliated organizations, or those of the publisher, the editors and the reviewers. Any product that may be evaluated in this article, or claim that may be made by its manufacturer, is not guaranteed or endorsed by the publisher.

## References

[ref1] Ahmad P. Hashem A. Abd-Allah E. F. Alqarawi A. John R. Egamberdieva D.. (2015). Role of *Trichoderma harzianum* in mitigating NaCl stress in Indian mustard (*Brassica juncea* L) through antioxidative defense system. Front. Plant Sci. 6:868. doi: 10.3389/fpls.2015.00868, PMID: 26528324 PMC4604702

[ref2] Akutse K. V. Maniania N. K. Fiaboe K. K. M. Berg J. V. Ekesi S. (2013). Endophytic colonization of *Vicia faba* and *Phaseolus vulgaris* (Fabaceae) by fungal pathogens and their effects on the life-history parameters of *Liriomyza huidobrensis* (Diptera: Agromyzidae). Fungal Ecol. 6, 293–301. doi: 10.1016/j.funeco.2013.01.003

[ref3] Almario J. Jeena G. Wunder J. Langen G. Zuccaro A. Coupland G.. (2017). Root-associated fungal microbiota of nonmycorrhizal *Arabis alpina* and its contribution to plant phosphorus nutrition. PNAS 114, E9403–E9412. doi: 10.1073/pnas.1710455114 28973917 PMC5676915

[ref4] Altomare C. Norvell W. A. Björkman T. Harman G. E. (1999). Solubilization of phosphates and micronutrients by the plant-growth-promoting and biocontrol fungus *Trichoderma harzianum* Rifai 1295-22. Appl. Environ. Microbiol. 65, 2926–2933. doi: 10.1128/AEM.65.7.2926-2933.1999, PMID: 10388685 PMC91438

[ref5] Aroca R. Alguacil M. M. Vernieri P. Ruiz-Lozano J. M. (2008). Plant responses to drought stress and exogenous ABA application are modulated differently by mycorrhizationin tomato and an ABA-deficient mutant (Sitiens). Microb. Ecol. 56, 704–719. doi: 10.1007/s00248-008-9390-y, PMID: 18443845

[ref6] Ashraf M. Foolad M. A. (2007). Improving plant abiotic-stress resistance by exogenous application of osmoprotectants glycine betaine and proline. Environ. Exp. Bot. 59, 206–216. doi: 10.1016/j.envexpbot.2005.12.006

[ref7] Atia M. A. M. Abdeldaym E. A. Abdelsattar M. Ibrahim D. S. S. Saleh I. Abd Elwahab M.. (2019). *Piriformospora indica* promotes cucumber tolerance against root-knot nematode by modulating photosynthesis and innate responsive genes. Saudi J. Biol. Sci. 27, 279–287. doi: 10.1016/j.sjbs.2019.09.007 31889848 PMC6933207

[ref8] Aziz L. Hamayun M. Rauf M. Iqbal A. Husssin A. Khan S. A.. (2021). *Aspergillus Flavus* reprogrammed morphological and chemical attributes of *Solanum lycopersicum* through *SlGSH1* and *SlPCS1* genes modulation under heavy metal stress. J. Plant Interact. 16, 104–115. doi: 10.1080/17429145.2021.1903105

[ref9] Baltruschat H. Fodor J. Harrach B. D. Niemczyk E. Barna B. Gullner G.. (2008). Salt tolerance of barley induced by the root endophyte *Piriformospora indica* is associated with a strong increase in anti-oxidants. New Phytol. 180, 501–510. doi: 10.1111/j.1469-8137.2008.02583.x, PMID: 18681935

[ref10] Bastias D. A. Martínez-Ghersa M. A. Ballaré C. L. Gundel P. E. (2017). *Epichloë* fungal endophytes and plant defenses: not just alkaloids. Trends Plant Sci. 22, 939–948. doi: 10.1016/j.tplants.2017.08.005, PMID: 28923242

[ref11] Berthelot C. Leyval C. Foulon J. Chalot M. Blaudez D. (2016). Plant growth promotion, metabolite production and metal tolerance of dark septate endophytes isolated from metal-polluted poplar phytomanagement sites. FEMS Microbiol. Ecol. 92:fiw144. doi: 10.1093/femsec/fiw144, PMID: 27364359

[ref12] Bilal S. Shahzad R. Imrana M. Jan R. Kim K. M. Lee I. J. (2020). Synergistic association of endophytic fungi enhances *Glycine max* L. resilience to combined abiotic stresses: heavy metals, high temperature and drought stress. Ind. Crop. Prod. 143:111931. doi: 10.1016/j.indcrop.2019.111931

[ref13] Bononi L. Chiaramonte J. B. Pansa C. C. Moitinho M. A. Melo I. S. (2020). Phosphorus-solubilizing *Trichoderma* spp. From Amazon soils improve soybean plant growth. Sci. Rep. 10, 1–13. doi: 10.1038/s41598-020-59793-8 32071331 PMC7028723

[ref14] Bonos S. A. Wilson M. M. Meyer W. A. Funk C. R. (2005). Suppression of redthread in fine fescues through endophyte-mediated resistance. Appl. Netw. Sci. 10:1094. doi: 10.1094/ATS-2005-0725-01-RS

[ref15] Brotman Y. Landau U. Cuadros-Inostroza A. Takayuki T. Fernie A. R. Chet I.. (2013). Trichoderma-plant root colonization: escaping early plant defense responses and activation of the antioxidant machinery for saline stress tolerance. PLOS Pathog. 9:e1003221. doi: 10.1371/journal.ppat.1003221, PMID: 23516362 PMC3597500

[ref16] Brundrett M. C. (2006). “Understanding the roles of multifunctional mycorrhizal and endophytic fungi,” in Microbial Root Endophytes. Soil Biology. *Vol*. 9. eds. Schulz B. J. E. Boyle C. J. C. Sieber T. N. (Berlin, Heidelberg: Springer), 281–298.

[ref17] Bu N. Li X. Li Y. Ma C. Ma L. Zhang C. (2012). Effects of Na2CO3 stress on photosynthesis and antioxidative enzymes in endophyte infected and non-infected rice. Ecotoxicol. Environ. Saf. 78, 35–40. doi: 10.1016/j.ecoenv.2011.11.007, PMID: 22138149

[ref18] Chaoui A. Ferjani E. E. (2005). Effects of cadmium and copper on antioxidant capacities, lignification and auxin degradation in leaves of pea (*Pisum sativum* L.) seedlings. Plant Biol. Pathol. 328, 23–31. doi: 10.1016/j.crvi.2004.10.001 15714877

[ref19] Chen Y. Peng Y. Dai C. C. Ju Q. (2011). Biodegradation of 4-hydroxybenzoic acid by *Phomopsis liquidambari*. Appl. Soil Ecol. 51, 102–110. doi: 10.1016/j.apsoil.2011.09.004

[ref20] Christian N. Herre E. A. Clay K. (2019). Foliar endophytic fungi alter patterns of nitrogen uptake and distribution in *Theobroma cacao*. New Phytol. 222, 1573–1583. doi: 10.1111/nph.15693, PMID: 30664252

[ref21] Chutipaijit S. Suriyan C. U. Sompornpailin K. (2009). Differential accumulations of proline and flavonoids in indica rice varieties against salinity. Pak. J. Bot. 41, 2497–2506.

[ref22] Clarke B. B. White J. F. J. Hurley R. H. Torres M. S. Sun S. Huff D. R. (2006). Endophyte-mediated suppression of dollar spot disease in fine fescues. Plant Dis. 90, 994–998. doi: 10.1094/PD-90-0994, PMID: 30781289

[ref23] Clay K. Holah J. (1999). Fungal endophyte symbiosis and plant diversity in successional fields. Science 285, 1742–1745. doi: 10.1126/science.285.5434.1742, PMID: 10481011

[ref24] Costa L. R. C. Azevedo J. L. Pereira J. O. Vieira M. L. C. Labate C. A. (2000). Symptomless infection of banana and maize by endophytic fungi impairs photosynthetic efficiency. New Phytol. 147, 609–615. doi: 10.1046/j.1469-8137.2000.00722.x 33862932

[ref25] Dai C. C. Xie H. Wang X. X. Li P. D. Li Y. L. Zhang T. L. (2010). The effects of intercropping with medicinal plants and addition of endophytic fungi on soil microflora and peanut yield. Acta Ecologica Sinica 30, 2105–2111.

[ref26] Dastogeer K. M. G. (2018). Influence of fungal endophytes on plant physiology is more pronounced under stress than well-watered conditions: a meta-analysis. Planta 248, 1403–1416. doi: 10.1007/s00425-018-2982-y, PMID: 30121874

[ref28] Del Rio D. Stewart A. J. Pellegrini N. (2005). A review of recent studies on malondialdehyde as toxic molecule and biological marker of oxidative stress. Nutr. Metab. Cardiovasc. Dis. 15, 316–328. doi: 10.1016/j.numecd.2005.05.003 16054557

[ref27] De Santiago A. Quintero J. M. Avilés M. Delgado A. (2009). Effect of *Trichoderma asperellum* strain T34 on iron nutrition in white lupin. Soil Biol. Biochem. 41, 2453–2459. doi: 10.1016/j.soilbio.2009.07.033

[ref30] Deshmukh P. S. Sairam R. K. Shukla D. S. (1991). Measurement of ion leakage as a screening technique for drought resistance in wheat genotypes. Indian J. Plant Physiol. 35, 89–91.

[ref31] Diene O. Wang W. Narisawa K. (2013). *Pseudosigmoidea ibarakiensis* sp. Nov., a dark septate endophytic fungus from a cedar forest in Ibaraki, Japan. Microbes Environ. 28, 381–387. doi: 10.1264/jsme2.ME13002, PMID: 24005845 PMC4070967

[ref32] Dietz K. J. Heber U. (1984). Rate-limiting factors in leaf photosynthesis. I. Carbon fluxes in the Calvin cycle. Biochim. Biophys. Acta Biomembr. 767, 432–443. doi: 10.1016/0005-2728(84)90041-0

[ref33] Dietz K. J. Heber U. (1986). Light and CO2 limitation of photosynthesis and states of the reactions regenerating ribulose 1, 5-bisphosphate or reducing 3-phosphoglycerate. Biochim. Biophys. Acta Biomembr. 848, 392–401. doi: 10.1016/0005-2728(86)90215-X

[ref34] Foyer C. H. Noctor G. (2011). Ascorbate and glutathione: the heart of the redox hub. Plant Physiol. 155, 2–18. doi: 10.1104/pp.110.167569, PMID: 21205630 PMC3075780

[ref35] Gange A. C. Koricheva J. Currie A. F. Jaber L. R. Vidal S. (2019). Meta-analysis of the role of entomopathogenic and unspecialized fungal endophytes as plant bodyguards. New Phytol. 223, 2002–2010. doi: 10.1111/nph.15859, PMID: 31002383 PMC6766880

[ref36] Geng Y. Wu R. Wee C. W. Xie F. Wei X. Chan P. M.. (2013). A spatio-temporal understanding of growth regulation during the salt stress response in *Arabidopsis*. Plant Cell 25, 2132–2154. doi: 10.1105/tpc.113.112896, PMID: 23898029 PMC3723617

[ref37] Gill S. S. Tuteja N. (2010). Reactive oxygen species and antioxidant machinery in abiotic stress tolerance in crop plants. Plant Physiol. Biochem. 48, 909–930. doi: 10.1016/j.plaphy.2010.08.016, PMID: 20870416

[ref38] Guo H. P. Hong C. T. Chen X. M. Xu Y. X. Liu Y. Jiang D. A.. (2016). Different growth and physiological responses to cadmium of the three *Miscanthus* species. PLoS One 11:e0153475. doi: 10.1371/journal.pone.0153475, PMID: 27070918 PMC4829268

[ref39] Hamayun M. Hussain A. Khan S. A. Kim H.-Y. Khan A. L. Waqas M.. (2017). Gibberellins producing endophytic fungus *Porostereum spadiceum* AGH786 rescues growth of salt affected soybean. Front. Microbiol. 8:686. doi: 10.3389/fmicb.2017.00686, PMID: 28473818 PMC5397423

[ref501] Hamilton C. E. Dowling T. E. Faeth S. H. (2010). Hybridization in endophyte symbionts alters host response to moisture and nutrient treatments. Microbial. Ecol. 59, 768–775. doi: 10.1007/s00248-009-9606-9, PMID: 19921327

[ref40] Hamilton C. E. Bauerle T. L. (2012). A new currency for mutualism? Fungal endophytes alter antioxidant activity in hosts responding to drought. Fungal Divers. 54, 39–49. doi: 10.1007/s13225-012-0156-y

[ref41] Harman G. E. Howell C. R. Viterbo A. Chet I. Lorito M. (2004). Trichoderma species opportunistic, avirulent plant symbionts. Nat. Rev. Microbiol. 2, 43–56. doi: 10.1038/nrmicro797, PMID: 15035008

[ref42] Hasanuzzaman M. Nahar K. Anee T. I. Fujita M. (2017). Glutathione in plants: biosynthesis and physiological role in environmental stress tolerance. Physiol. Mol. Biol. Plants 23, 249–268. doi: 10.1007/s12298-017-0422-2, PMID: 28461715 PMC5391355

[ref43] Hashem A. Abd-Allah E. Alqarawi A. Al Huqail A. A. Egamberdieva D. (2014). Alleviation of abiotic salt stress in *Ochradenus baccatus* (Del.) by *Trichoderma hamatum* (Bonord.) Bainier. J. Plant Interact. 9, 857–868. doi: 10.1080/17429145.2014.983568

[ref44] Herrera-Medina M. J. Steinkellner S. Vierheilig H. Bote J. A. O. Garrido J. M. G. (2007). Abscisic acid determines arbuscule development and functionality in the tomato arbuscular mycorrhiza. New Phytol. 175, 554–564. doi: 10.1111/j.1469-8137.2007.02107.x, PMID: 17635230

[ref45] Hill P. W. Broughton R. Bougoure J. Havelange W. Newsham K. K. Grant H.. (2019). Angiosperm symbioses with non-mycorrhizal fungal partners enhance N acquisition from ancient organic matter in a warming maritime Antarctic. Ecol. Lett. 22, 2111–2119. doi: 10.1111/ele.13399, PMID: 31621153 PMC6899649

[ref46] Hiruma K. Gerlach N. Sacristán S. Nakano R. T. Hacquard S. Kracher B.. (2016). Root endophyte *Colletotrichum tofieldiae* confers plant fitness benefits that are phosphate status dependent. Cell 165, 464–474. doi: 10.1016/j.cell.2016.02.028, PMID: 26997485 PMC4826447

[ref47] Hiruma K. Kobae Y. Toju H. (2018). Beneficial associations between Brassicaceae plants and fungal endophytes under nutrient-limiting conditions: evolutionary origins and host–symbiont molecular mechanisms. Curr. Opin. Plant Biol. 44, 145–154. doi: 10.1016/j.pbi.2018.04.009, PMID: 29738938

[ref48] Hoysted G. Jacob A. S. Kowal J. Giesemann P. Bidartondo M. I. Duckett J. G.. (2019). Mucoromycotina fine root endophyte fungi form nutritional mutualisms with vascular plants. Plant Physiol. 181, 565–577. doi: 10.1104/pp.19.00729, PMID: 31358684 PMC6776871

[ref49] Inderjit Nilsen E. T. (2003). Bioassays and field studies for allelopathy in terrestrial plants: progress and problems. Crit. Rev. Plant Sci. 22, 221–238. doi: 10.1080/713610857

[ref50] Ismail Hamayun M. Hussain A. Iqbal A. Khan S. A. Ahmad A.. (2021). *Aspergillus foetidus* regulated the biochemical characteristics of soybean and sunflower under heat stress condition: role in sustainability. Sustainability 13:7159. doi: 10.3390/su13137159

[ref51] Ismail Hamayun M. Hussain A. Iqbal A. Khan S. A. Lee I. J. (2018). Endophytic fungus *Aspergillus japonicus* mediates host plant growth under normal and heat stress conditions. Biomed. Res. Int. 2018:7696831. doi: 10.1155/2018/7696831, PMID: 30627568 PMC6304497

[ref52] Ismail A. Takeda S. Nick P. (2014). Life and death under salt stress: same players, different timing? J. Exp. Bot. 65, 2963–2979. doi: 10.1093/jxb/eru159, PMID: 24755280

[ref53] Jaakola L. Hohtola A. (2010). Effect of latitude on flavonoid biosynthesis in plants. Plant Cell Environ. 33, 1239–1247. doi: 10.1111/j.1365-3040.2010.02154.x, PMID: 20374534

[ref54] Johnson L. J. Johnson R. D. Schardl C. L. Panaccione D. G. (2003). Identification of differentially expressed genes in the mutualistic association of tall fescue with *Neotyphodium coenophialum*. Physiol. Mol. Plant Pathol. 63, 305–317. doi: 10.1016/j.pmpp.2004.04.001

[ref55] Jumpponen A. Trappe M. (1998). Dark septate endophytes: a review of facultative biotrophic root-colonizing fungi. New Phytol. 140, 295–310. doi: 10.1046/j.1469-8137.1998.00265.x, PMID: 33862835

[ref56] Kaushal N. Gupta K. Bhandhari K. Kumar S. Thakur P. Nayyar H. (2011). Proline induces heat tolerance in chickpea (*Cicer arietinum* L.) plants by protecting vital enzymes of carbon and antioxidative metabolism. Physiol. Mol. Biol. Plants 17, 203–213. doi: 10.1007/s12298-011-0078-2, PMID: 23573011 PMC3550571

[ref57] Khan A. L. Hamayun M. Kim Y. H. Kang S. M. Lee I. J. (2011a). Ameliorative symbiosis of endophyte (*Penicillium funiculosum* LHL06) under salt stress elevated plant growth of *Glycine max* L. Plant Physiol. Biochem. 49, 852–861. doi: 10.1016/j.plaphy.2011.03.005 21458283

[ref58] Khan A. L. Hamayun M. Kim Y. H. Kang S. M. Lee J. H. Lee I. J. (2011b). Gibberellins producing endophytic *Aspergillus fumigatus* sp. LH02 influenced endogenous phytohormonal levels, isoflavonoids production and plant growth in salinity stress. Process Biochem. 46, 440–447. doi: 10.1016/j.procbio.2010.09.013

[ref59] Khan S. A. Hamayun M. Yoon H. Kim H. Y. Suh S. J. Hwang S. K.. (2008). Plant growth promotion and *Penicillium citrinum*. BMC Microbiol. 8:231. doi: 10.1186/1471-2180-8-231, PMID: 19099608 PMC2631606

[ref60] Khan A. L. Kang S. M. Dhakal K. H. Hussain J. Adnan M. Kim J. G.. (2013). Flavonoids and amino acid regulation in *Capsicum annuum* L. by endophytic fungi under different heat stress regimes. Sci. Hortic. 155, 1–7. doi: 10.1016/j.scienta.2013.02.028

[ref61] Khan A. R. Ullah I. Waqas M. Park G. S. Khan A. L. Hong S. J.. (2017). Host plant growth promotion and cadmium detoxification in *Solanum nigrum*, mediated by endophytic fungi. Ecotoxicol. Environ. Saf. 136, 180–188. doi: 10.1016/j.ecoenv.2016.03.014, PMID: 27931714

[ref62] Khan A. L. Waqas M. Lee I. J. (2015). Resilience of *Penicillium resedanum* LK6 and exogenous gibberellin in improving *Capsicum annuum* growth under abiotic stresses. J. Plant Res. 128, 259–268. doi: 10.1007/s10265-014-0688-1, PMID: 25537300

[ref63] Khare E. Mishra J. Arora N. K. (2018). Multifaceted interactions between endophytes and plant: developments and prospects. Front. Microbiol. 9:2732. doi: 10.3389/fmicb.2018.02732, PMID: 30498482 PMC6249440

[ref64] Khushdil F. Jan F. G. Jan G. Hamayun M. Iqbal A. Hussain A.. (2019). Salt stress alleviation in *Pennisetum glaucum* through secondary metabolites modulation by *Aspergillus terreus*. Plant Physiol. Biochem. 144, 127–134. doi: 10.1016/j.plaphy.2019.09.038, PMID: 31563093

[ref65] Kishor P. B. K. Sangam S. Amrutha R. N. Laxmi P. S. Naidu K. R. Rao K. R. S. S.. (2005). Regulation of proline biosynthesis, degradation, uptake and transport in higher plants: its implications in plant growth and abiotic stress tolerance. Curr. Sci. 88, 424–438.

[ref66] Koroch A. Juliani H. Bischoff J. Lewis E. Bills G. Simon J.. (2004). Examination of plant biotrophy in the scale insect parasitizing fungus *Dussiella tuberiformis*. Symbiosis 37, 267–280.

[ref67] Lacey L. A. Neven L. G. (2006). The potential of the fungus, *Muscodor albus*, as a microbial control agent of potato tuber moth (Lepidoptera: Gelechiidae) in stored potatoes. J. Invertebr. Pathol. 91, 195–198. doi: 10.1016/j.jip.2006.01.002, PMID: 16494898

[ref68] Lam C. K. Belanger F. C. White J. F. Jr. Daie J. (1995). Invertase activity in Epichloë/Acremonium fungal endophytes and its possible role in choke disease. Mycol. Res. 99, 867–873. doi: 10.1016/S0953-7562(09)80743-0

[ref69] Lamaoui M. Jemo M. Datla R. Bekkaoui F. (2018). Heat and drought stresses in crops and approaches for their mitigation. Front. Chem. 6:26. doi: 10.3389/fchem.2018.00026, PMID: 29520357 PMC5827537

[ref70] Leitão A. L. Enguita F. A. (2016). Gibberellins in *Penicillium* strains: challenges for endophyte-plant host interactions under salinity stress. Microbiol. Res. 183, 8–18. doi: 10.1016/j.micres.2015.11.004, PMID: 26805614

[ref71] Li X. Bua N. Li Y. Ma L. Xin S. Zhang L. (2012). Growth, photosynthesis and antioxidant responses of endophyte infected and non-infected rice under lead stress conditions. J. Hazard. Mater. 213–214, 55–61. doi: 10.1016/j.jhazmat.2012.01.052 22356744

[ref72] Li S. Hartman G. L. Boykin D. L. (2010). Aggressiveness of *Phomopsis longicolla* and other *Phomopsis* spp. On Soybean. Plant Dis. 94, 1035–1040. doi: 10.1094/PDIS-94-8-1035, PMID: 30743477

[ref73] Li C. Nan Z. Paul V. H. Dapprich P. Liu Y. (2004). A new Neotyphodium species symbiotic with drunken horse grass (*Achnatherum inebrians*) in China. Mycotaxon 90, 141–147.

[ref74] Li X. Zhou J. Xu R. S. Meng M. Yu X. Dai C. C. (2018). Auxin, cytokinin, and ethylene involved in rice N availability improvement caused by endophyte *Phomopsis liquidambari*. J. Plant Growth Regul. 37, 128–143. doi: 10.1007/s00344-017-9712-8

[ref75] Lubna A. S. Hamayuna M. Khan A. L. Waqas M. Khan M. A. Jan R.. (2018). Salt tolerance of *Glycine max* L. induced by endophytic fungus *Aspergillus flavus* CSH1, via regulating its endogenous hormones and antioxidative system. Plant Physiol. Biochem. 128, 13–23. doi: 10.1016/j.plaphy.2018.05.007, PMID: 29751251

[ref76] Lukešová T. Kohout P. Větrovský T. Vohník M. (2015). The potential of dark septate endophytes to form root symbioses with ectomycorrhizal and ericoid mycorrhizal middle European forest plants. PLoS One 10:e0124752. doi: 10.1371/journal.pone.0124752, PMID: 25905493 PMC4408093

[ref77] Mahmoud R. S. Narisawa K. (2013). A new fungal endophyte, *Scolecobasidium humicola*, promotes tomato growth under organic nitrogen conditions. PLoS One 8:e78746. doi: 10.1371/journal.pone.0078746, PMID: 24223848 PMC3815298

[ref78] Malinowski D. P. Alloush G. A. Belesky D. P. (2000). Leaf endophyte *Neotyphodium coenophialum* modifies mineral uptake in tall fescue. Plant Soil 227, 115–126. doi: 10.1023/A:1026518828237

[ref79] Mandyam K. Loughin T. Jumpponen A. (2010). Isolation and morphological and metabolic characterization of common endophytes in annually burned tallgrass prairie. Mycologia 102, 813–821. doi: 10.3852/09-212 20648749

[ref80] Marks S. Clay K. (1996). Physiological responses of *Festuca arundinacea* to fungal endophyte infection. New Phytol. 133, 727–733. doi: 10.1111/j.1469-8137.1996.tb01941.x

[ref81] Martinuz A. Schouten A. Sikora R. A. (2012). Systemically induced resistance and microbial competitive exclusion: implications on biological control. Phytopathology 102, 260–266. doi: 10.1094/PHYTO-04-11-0120, PMID: 21899390

[ref82] Mehmood A. Hussain A. Irshad M. Hamayun M. Iqbal A. Khan N. (2019). In vitro production of IAA by endophytic fungus *Aspergillus awamori* and its growth promoting activities in *Zea mays*. Symbiosis 77, 225–235. doi: 10.1007/s13199-018-0583-y

[ref83] Menkis A. Allmer J. Vasiliauskas R. Lygis V. Stenlid J. Finlay R. (2004). Ecology and molecular characterization of dark septate fungi from roots, living stems, coarse and fine woody debris. Mycol. Res. 108, 965–973. doi: 10.1017/S0953756204000668, PMID: 15449602

[ref84] Morse L. J. Day T. A. Faeth S. H. (2002). Effect of *Neotyphodium* endophyte infection on growth and leaf gas exchange of Arizona fescue under contrasting water availability regimes. Environ. Exp. Bot. 45, 256–268. doi: 10.1016/S0098-8472(02)00042-4

[ref86] Moy M. Belanger F. Duncan R. Freehoff A. Leary C. Meyer W.. (2000). Identification of epiphyllous mycelial nets on leaves of grasses infected by clavicipitaceous endophytes. Symbiosis 28, 291–302.

[ref87] Moy M. Li H. M. Sullivan R. White J. F. Belanger F. C. (2002). Endophytic fungal β-1, 6-glucanase expression in the infected host grass. Plant Physiol. 130, 1298–1308. doi: 10.1104/pp.010108, PMID: 12427996 PMC166650

[ref88] Newsham K. K. (1999). *Phialophora graminicola*, a dark septate fungus, is a beneficial associate of the grass *Vulpia ciliata* ssp. ambigua. New Phytol. 144, 517–524. doi: 10.1046/j.1469-8137.1999.00537.x, PMID: 33862854

[ref89] Newsham K. K. (2011). A meta-analysis of plant responses to dark septate root endophytes. New Phytol. 190, 783–793. doi: 10.1111/j.1469-8137.2010.03611.x, PMID: 21244432

[ref90] Newsham K. K. Upson R. Read D. J. (2009). Mycorrhizas and dark septate root endophytes in polar regions. Fungal Ecol. 2, 10–20. doi: 10.1016/j.funeco.2008.10.005 19495811

[ref92] Panaccione D. G. Cipoletti J. R. Sedlock A. B. Blemings K. P. Schardl C. L. Machado C.. (2006). Effects of ergot alkaloids on food preference and satiety in rabbits as assessed with gene-knockout endophytes in perennial ryegrass (*Lolium perenne*). J. Agric. Food Chem. 54, 4582–4587. doi: 10.1021/jf060626u, PMID: 16787001

[ref93] Pandey V. Ansari M. W. Tula S. Yadav S. Sahoo R. K. Shukla N.. (2016). Dose-dependent response of *Trichoderma harzianum* in improving drought tolerance in rice genotypes. Planta 243, 1251–1264. doi: 10.1007/s00425-016-2482-x, PMID: 26898554

[ref94] Pedraza R. O. Díaz C. T. Lavín P. Molina P. R. Atala C. Cerda J. G.. (2020). Root endophytic *Penicillium* promotes growth of Antarctic vascular plants by enhancing nitrogen mineralization. Extremophiles 24, 721–732. doi: 10.1007/s00792-020-01189-7 32699913

[ref95] Peterson R. D. Theimer C. A. Wu H. Feigon J. (2004). New applications of 2D filtered/edited NOESY for assignment and structure elucidation of RNA and RNA-protein complexes. J. Biomol. NMR 28, 59–67. doi: 10.1023/B:JNMR.0000012861.95939.05, PMID: 14739639

[ref96] Pietro-Souza W. Pereira F. C. Mello I. S. Stachack F. F. F. Terezo A. J. Cunha C. N.. (2020). Mercury resistance and bioremediation mediated by endophytic fungi. Chemosphere 240:124874. doi: 10.1016/j.chemosphere.2019.124874, PMID: 31546184

[ref97] Ramos P. Rivas N. Pollmann S. Casati P. Molina-Montenegro M. A. (2018). Hormonal and physiological changes driven by fungal endophytes increase Antarctic plant performance under UV-B radiation. Fungal Ecol. 34, 76–82. doi: 10.1016/j.funeco.2018.05.006

[ref98] Ranieri E. Moustakas K. Barbafieri M. Ranieri A. C. Herrera-Melian J. A. Petrella A.. (2019). Phytoextraction technologies for mercury- and chromium-contaminated soil: a review. J. Chem. Technol. Biotechnol. 95, 317–327. doi: 10.1002/jctb.6008

[ref99] Richardson M. D. Hoveland C. S. Bacon C. W. (1993). Photosynthesis and stomatal conductance of symbiotic and nonsymbiotic tall fescue. Crop Sci. 33, 145–149. doi: 10.2135/cropsci1993.0011183X003300010026x

[ref100] Rocha A. C. S. Garcia D. Uetanabaro A. P. T. Carneiro R. T. O. Araújo I. S. Mattos C. R. R.. (2011). Foliar endophytic fungi from *Hevea brasiliensis* and their antagonism on *Microcyclusulei*. Fungal Divers. 47, 75–84. doi: 10.1007/s13225-010-0044-2

[ref101] Rodriguez R. J. White J. F. J. Arnold A. E. Redman A. R. S. (2009). Fungal endophytes: diversity and functional roles. New Phytol. 182, 314–330. doi: 10.1111/j.1469-8137.2009.02773.x, PMID: 19236579

[ref102] Royles J. Griffiths H. (2015). Invited review: climate change impacts in polar regions: lessons from Antarctic moss bank archives. Glob. Chang. Biol. 21, 1041–1057. doi: 10.1111/gcb.12774 25336089

[ref103] Rozpądek P. Wężowicz K. Nosek M. Ważny R. Tokarz K. Lembicz M.. (2015). The fungal endophyte *Epichloë typhina* improves photosynthesis efficiency of its host orchard grass (*Dactylis glomerata*). Planta 242, 1025–1035. doi: 10.1007/s00425-015-2337-x, PMID: 26059605 PMC4560772

[ref104] Sadeghi F. Samsampour D. Seyahooei M. A. Bagheri A. Soltani J. (2019). Fungal endophytes alleviate drought-induced oxidative stress in mandarin (*Citrus reticulata* L.): toward regulating the ascorbate–glutathione cycle. Sci. Hortic. 261:108991. doi: 10.1016/j.scienta.2019.108991

[ref105] Saikkonen K. Saari S. Helander M. (2010). Defensive mutualism between plants and endophytic fungi? Fungal Divers. 41, 101–113. doi: 10.1007/s13225-010-0023-7

[ref106] Samolski I. Rincon A. M. Pinzón L. M. Viterbo A. Monte E. (2012). The qid74 gene from *Trichoderma harzianum* has a role in root architecture and plant biofertilization. Microbiology 158, 129–138. doi: 10.1099/mic.0.053140-0, PMID: 21948047

[ref107] Scholes J. D. Lee P. J. Horton P. Lewis D. H. (1994). Invertase: understanding changes in the photosynthetic and carbohydrate metabolism of barley leaves infected with powdery mildew. New Phytol. 126, 213–222. doi: 10.1111/j.1469-8137.1994.tb03939.x

[ref108] Schulz B. Boyle C. (2005). The endophytic continuum. Mycol. Res. 109, 661–686. doi: 10.1017/S095375620500273X, PMID: 16080390

[ref109] Shahabivand S. Parvaneh A. Aliloo A. A. (2017). Root endophytic fungus *Piriformospora indica* affected growth, cadmium partitioning and chlorophyll fluorescence of sunflower under cadmium toxicity. Ecotoxicol. Environ. Saf. 145, 496–502. doi: 10.1016/j.ecoenv.2017.07.064, PMID: 28783599

[ref110] Shahollari B. Varma A. Oelmüller R. (2005). Expression of a receptor kinase in *Arabidopsis* roots is stimulated by the basidiomycete *Piriformospora indica* and the protein accumulates in triton X-100 insoluble plasma membrane microdomains. J. Plant Physiol. 162, 945–958. doi: 10.1016/j.jplph.2004.08.012, PMID: 16146321

[ref111] Sharma S. B. Sayyed R. Z. Trivedi M. H. Gobi T. A. (2013). Phosphate solubilizing microbes: sustainable approach for managing phosphorus deficiency in agricultural soils. Springerplus 2:587. doi: 10.1186/2193-1801-2-587, PMID: 25674415 PMC4320215

[ref112] Sherameti I. Shahollari B. Venus Y. Altschmied L. Varma A. Oelmüller R. (2005). The endophytic fungus *Piriformospora indica* stimulates the expression of nitrate reductase and the starch-degrading enzyme glucan-water dikinase in tobacco and *Arabidopsis* roots through a homeodomain transcription factor that binds to a conserved motif in their promoters. J. Biol. Chem. 280, 26241–26247. doi: 10.1074/jbc.M500447200 15710607

[ref502] Shi Y. Xie H. Cao L. (2017). Effects of Cd- and Pb-resistant endophytic fungi on growth and phytoextraction of Brassica napus in metal-contaminated soils. Environ. Sci. Pollut. Res. 24, 417–426. doi: 10.1007/s11356-016-7693-y, PMID: 27726080

[ref113] Shishido M. Sato K. Yoshida N. Tsukui R. Usami T. (2010). PCR-based assays to detect and quantify *Phomopsis sclerotioides* in plants and soil. J. Gen. Plant Pathol. 76, 21–30. doi: 10.1007/s10327-009-0209-6

[ref114] Singh S. P. Pandey S. Mishra N. Giri V. P. Mahfooz S. Bhattacharya A.. (2019). Supplementation of *Trichoderma* improves the alteration of nutrient allocation and transporter genes expression in rice under nutrient deficiencies. Plant Physiol. Biochem. 143, 351–363. doi: 10.1016/j.plaphy.2019.09.015, PMID: 31541990

[ref115] Smith S. E. Read D. J. (2008). Mycorrhizal Symbiosis. 3rd *Edn*. Academic Press.

[ref116] Sondergaard T. E. Schulz A. Palmgren M. G. (2004). Energization of transport processes in plants. Roles of the plasma membrane H^+^-ATPase. Plant Physiol. 136, 2475–2482. doi: 10.1104/pp.104.048231, PMID: 15375204 PMC523315

[ref117] Song M. Li X. Saikkonen K. Li C. Nan Z. (2015). An asexual *Epichloë* endophyte enhances waterlogging tolerance of *Hordeum brevisubulatum*. Fungal Ecol. 13, 44–52. doi: 10.1016/j.funeco.2014.07.004

[ref118] Soto-Barajas M. C. Zabalgogeazcoa I. Fuertes J. G. Blanco V. G. Vázquez-de-Aldana B. R. (2016). *Epichloë* endophytes affect the nutrient and fiber content of *Lolium perenne* regardless of plant genotype. Plant Soil 405, 265–277. doi: 10.1007/s11104-015-2617-z

[ref119] Spatafora J. W. Sung G. H. Sung J. M. Hywel-Jones N. L. White J. F. (2007). Phylogenetic evidence for an animal pathogen origin of ergot and the grass endophytes. Mol. Ecol. 16, 1701–1711. doi: 10.1111/j.1365-294X.2007.03225.x, PMID: 17402984

[ref120] Spiering M. J. Greer D. H. Schmid J. (2006). Effects of the fungal endophyte, *Neotyphodium lolii*, on net photosynthesis and growth rates of perennial ryegrass (*Lolium perenne*) are independent of in planta endophyte. Ann. Bot. 98, 379–387. doi: 10.1093/aob/mcl108, PMID: 16735403 PMC2803460

[ref121] Su C. L. Zhang F. Sun K. Zhang W. Dai C. C. (2019). Fungal endophyte *Phomopsis liquidambari* improves iron and molybdenum nutrition uptake of peanut in consecutive monoculture soil. J. Soil Sci. Plant Nutr. 19, 71–80. doi: 10.1007/s42729-019-0011-2

[ref122] Sun C. Johnson J. Cai D. Sherameti I. Oelmüeller R. Lou B. (2010). *Piriformospora indica* confers drought tolerance in Chinese cabbage leaves by stimulating antioxidant enzymes, the expression of drought-related genes and the plastid-localized CAS protein. J. Plant Physiol. 167, 1009–1017. doi: 10.1016/j.jplph.2010.02.013, PMID: 20471134

[ref123] Surono Narisawa K. (2017). The dark septate endophytic fungus *Phialocephala fortinii* is a potential decomposer of soil organic compounds and a promoter of *Asparagus officinalis* growth. Fungal Ecol. 28, 1–10. doi: 10.1016/j.funeco.2017.04.001

[ref124] Tanaka A. Christensen M. J. Takemoto D. Pyoyun P. Scott B. (2006). Reactive oxygen species play a role in regulating a fungus perennial ryegrass mutualistic interaction. Plant Cell 18, 1052–1066. doi: 10.1105/tpc.105.039263, PMID: 16517760 PMC1425850

[ref125] Torres M. S. White J. F. J. Bischoff J. F. (2007). *Hypocrella panamensis* sp. Nov (Clavicipitaceae Hypocreales): evaluation on the basis of morphological and molecular characters. Mycol. Res. 111, 317–323. doi: 10.1016/j.mycres.2007.01.005, PMID: 17368009

[ref126] Upson R. Newsham K. K. Read D. J. (2008). Root-fungal associations of *Colobanthus quitensis* and *Deschampsia antarctica* in the maritime and subantarctic. Arct. Antarct. Alp. Res. 40, 592–599. doi: 10.1657/1523-0430(07-057)[UPSON]2.0.CO;2

[ref127] Upson R. Read D. J. Newsham K. K. (2009). Nitrogen form influences the response of *Deschampsia antarctica* to dark septate root endophytes. Mycorrhiza 20, 1–11. doi: 10.1007/s00572-009-0260-3, PMID: 19495811

[ref128] Usuki F. Narisawa K. (2007). A mutualistic symbiosis between a dark septate endophytic fungus, *Heteroconium chaetospira*, and a nonmycorrhizal plant, Chinese cabbage. Mycologia 99, 175–184. doi: 10.1080/15572536.2007.11832577 17682770

[ref129] Vázquez-de-Aldana B. R. Criado B. G. Tavera S. V. Zabalgogeazcoa I. (2013). Fungal endophyte (*Epichloë festucae*) alters the nutrient content of *Festuca rubra* regardless of water availability. PLoS One 8:e84539. doi: 10.1371/journal.pone.0084539 24367672 PMC3867530

[ref130] Vergara C. Araujo K. E. C. Alves L. S. de Souza S. R. Santos L. A. Santa-Catarina C.. (2018). Contribution of dark septate fungi to the nutrient uptake and growth of rice plants. Braz. J. Microbiol. 49, 67–78. doi: 10.1016/j.bjm.2017.04.010, PMID: 28888828 PMC5790585

[ref131] Vergara C. Araujo K. E. C. Sperandio M. V. L. Santos L. A. Urquiaga S. Zilli J. E. (2019). Dark septate endophytic fungi increase the activity of proton pumps, efficiency of 15N recovery from ammonium sulphate, N content, and micronutrient levels in rice plants. Braz. J. Microbiol. 50, 825–838. doi: 10.1007/s42770-019-00092-4, PMID: 31090019 PMC6863334

[ref132] Vergara C. Araujo K. E. C. Urquiaga S. Schultz N. de Carvalho Balieiro F. Medeiros P. S.. (2017). Dark septate endophytic fungi help tomato to acquire nutrients from ground plant material. Front. Microbiol. 8:2437. doi: 10.3389/fmicb.2017.02437, PMID: 29312163 PMC5732191

[ref133] Wang J. L. Li T. Liu G. Y. Smith J. M. Zhao Z. W. (2016). Unraveling the role of dark septateendophyte (DSE) colonizing maize (*Zea mays*) under cadmium stress: physiological, cytological and genic aspects. Sci. Rep. 6:22028. doi: 10.1038/srep22028, PMID: 26911444 PMC4766571

[ref134] Wang J. Nan Z. Christensen M. J. Zhang X. Tian P. Zhang Z.. (2018). Effect of *Epichloë gansuensis* endophyte on the nitrogen metabolism, nitrogen use efficiency, and stoichiometry of *Achnatherum inebrians* under nitrogen LIMITATION. J. Agric. Food Chem. 66, 4022–4031. doi: 10.1021/acs.jafc.7b06158, PMID: 29648822

[ref135] Wani S. H. Kumar V. Shriram V. Sah S. K. (2016). Phytohormones and their metabolic engineering for abiotic stress tolerance in crop plants. Crop J. 4:162176. doi: 10.1016/j.cj.2016.01.010

[ref136] Waqas M. Khan A. L. Kamran M. Hamayun M. Kang S. M. Kim Y. H.. (2012). Endophytic fungi produce gibberellins and indoleacetic acid and promotes host-plant growth during stress. Molecules 17, 10754–10773. doi: 10.3390/molecules170910754, PMID: 22960869 PMC6268353

[ref137] Waqas M. Khan A. L. Kang S. M. Kim Y. H. Lee I. J. (2014). Phytohormone-producing fungal endophytes and hardwood-derived biochar interact to ameliorate heavymetal stress in soybeans. Biol. Fertil. Soils 50, 1155–1167. doi: 10.1007/s00374-014-0937-4

[ref138] Wei Y. F. Li T. Li L. F. Wang J. L. Cao G. H. Zhao Z. W. (2016). Functional and transcript analysis of a novel metal transporter gene *EpNramp* from a dark septate endophyte (*Exophiala pisciphila*). Ecotoxicol. Environ. Saf. 124, 363–368. doi: 10.1016/j.ecoenv.2015.11.008, PMID: 26595509

[ref139] White J. J. F. Torres M. S. (2010). Is plant endophyte-mediated defensive mutualism the result of oxidative stress protection? Physiol. Plant. 138, 440–446. doi: 10.1111/j.1399-3054.2009.01332.x, PMID: 20028480

[ref140] Wichern F. Lobe I. Amelung W. Müller T. Joergensen R. G. Buerkert A. (2004). Changes in amino acid enantiomers and microbial performance in soils from a subtropical mountain oasis in Oman abandoned for different periods. Biol. Fertil. Soils 39, 398–406. doi: 10.1007/s00374-004-0727-5

[ref141] Wu C. Li B. Wei Q. Pan R. Zhang W. (2019). Endophytic fungus *Serendipita indica* increased nutrition absorption and biomass accumulation in *Cunninghamia lanceolata* seedlings under low phosphate. Acta Ecol. Sin. 39, 21–29. doi: 10.1016/j.chnaes.2018.06.005

[ref142] Xia C. Li N. Zhang X. Feng Y. Christensen M. J. Nan Z. (2016). An *Epichloë* endophyte improves photosynthetic ability and drymatter production of its host *Achnatherum inebrians* infected by *Blumeria graminis* under various soil water conditions. Fungal Ecol. 22, 26–34. doi: 10.1016/j.funeco.2016.04.002

[ref143] Yadav V. Kumar M. Deep D. K. Kumar H. Sharma R. Tripathi T.. (2010). A phosphate transporter from the root endophytic fungus *Piriformospora indica* plays a role in phosphate transport to the host plant. J. Biol. Chem. 285, 26532–26544. doi: 10.1074/jbc.M110.111021, PMID: 20479005 PMC2924090

[ref144] Yan N. Wang X. Q. Xu X. F. Guo D. P. Wang Z. D. Zhang J. Z.. (2013). Plant growth and photosynthetic performance of *Zizania latifolia* are altered by endophytic *Ustilago esculenta* infection. Physiol. Mol. Plant Pathol. 83, 75–83. doi: 10.1016/j.pmpp.2013.05.005

[ref145] Yang B. Ma H. Y. Wang X. Jia Y. Hu J. Li X.. (2014). Improvement of nitrogen accumulation and metabolism in rice (*Oryza sativa* L.) by the endophyte *Phomopsis liquidambari*. Plant Physiol. Biochem. 82, 172–182. doi: 10.1016/j.plaphy.2014.06.002, PMID: 24972305

[ref146] Yasmeen R. Siddiqui Z. (2017). Physiological responses of crop plants against *Trichoderma Harzianum* in saline environment. Acta Bot. Croat. 76, 154–162.

[ref147] Yihui B. Zhouying X. U. Yurong Y. Haihan Z. Hui C. Ming T. (2017). Effect of dark septate endophytic fungus *Gaeumannomyces cylindrosporus* on plant growth, photosynthesis and Pb tolerance of maize (*Zea mays* L.). Pedosphere 27, 283–292. doi: 10.1016/S1002-0160(17)60316-3

[ref148] Yoneyama T. Ito O. Engelaar W. (2003). Uptake, metabolism and distribution of nitrogen in crop plants traced by enriched and natural ^15^N: progress over the last 30 years. Phytochem. Rev. 2, 121–132. doi: 10.1023/B:PHYT.0000004198.95836.ad

[ref149] You Y. H. Yoon H. Kang S. M. Shin J. H. Choo Y. S. Lee I. J.. (2012). Fungal diversity and plant growth promotion of endophytic fungi from six halophytes in Suncheon Bay. J. Microbiol. Biotechnol. 22, 1549–1556. doi: 10.4014/jmb.1205.05010, PMID: 23124347

